# Urease promotes pH homeostasis and growth of *Staphylococcus aureus* in skin-like conditions

**DOI:** 10.1128/jb.00208-25

**Published:** 2025-09-08

**Authors:** Flavia G. Costa, Alexander R. Horswill

**Affiliations:** 1 Department of Immunology and Microbiology, University of Colorado Anschutz Medical Campus 129263, Aurora, Colorado, USA; 2 Department of Veterans Affairs, Eastern Colorado Healthcare System, Aurora, Colorado, USA; The Ohio State University, Columbus, Ohio, USA

**Keywords:** acid tolerance, pH homeostasis, skin colonization, *Staphylococcus aureus*, urease, microbial physiology

## Abstract

*Staphylococcus aureus* is a Gram-positive opportunistic pathogen and a leading cause of skin and soft tissue infections (SSTIs). *S. aureus* colonizes approximately 20–30% of the population and transiently colonizes the skin. Although skin colonization is an SSTI risk factor, our understanding of *S. aureus* requirements to persist in this environment is still developing. The human skin is acidic, with a ubiquitous distribution of eccrine glands, which secrete metabolites, such as urea onto the skin surface. Many pathogens break down urea into ammonia and carbon dioxide by the Ni(II)-dependent enzyme urease as an acid resistance mechanism. *S. aureus* relies on urea metabolism for pH homeostasis in the murine kidney, but a role for this process on skin has not been shown. Using an *in vitro* model of the human skin surface environment, we investigated the role of urease in the methicillin-resistant USA300 strain *S. aureus* subsp. *aureus* (MRSA) LAC. We observe that urease contributes to an increase in extracellular pH and production of extracellular ammonium, which results in ammonium becoming a shared good in a mixed culture. We also demonstrate that the putative urea transporter SAUSA300_2237 (*ureT*) is required for optimal extracellular ammonia generation. Finally, we show that the urease inhibitor fluorofamide effectively inhibits *S. aureus* urease activity. This work identifies a role for urea metabolism during *S. aureus* skin colonization and offers the first published characterization of the *S. aureus* putative urea transporter, UreT.

**IMPORTANCE:**

The human skin is a unique microbial environment that has been challenging to faithfully model in the laboratory. Here, we utilize a recently developed media recipe incorporating metabolites measured from healthy human skin to assess the role of urease in *Staphylococcus aureus* pH homeostasis in the skin environment. These data provide new insights into the role of urea metabolism in *S. aureus* pH homeostasis during skin colonization. We also describe the first characterization of transporter SAUSA300_2237 (*ureT*) and demonstrate its role in urea metabolism. These insights advance our understanding of this metabolic pathway in *S. aureus*.

## INTRODUCTION

*Staphylococcus aureus* (*S. aureus*) is an opportunistic pathogen that can cause infections in nearly every body site in humans (1). *S. aureus* asymptomatically colonizes approximately 20–30% of the human population and is most frequently detected in the anterior nares of colonized persons (2–4). Using previously reported data, it was estimated that carriage of *S. aureus* in the anterior nares is correlated to increased frequency of detection of *S. aureus* on other parts of the body (4). *S. aureus* is often detected on healthy skin in these carriers, supporting that *S. aureus* is able to colonize healthy skin, thus highlighting a need to understand the factors required for colonization and persistence in this environment (5).

The human skin is a unique and metabolically challenging environment for microorganisms, in part due to the acidic pH that distinguishes human skin from the skin pH of other mammals (6). The pH of healthy human skin has been measured between 4.1 and 5.8 at the stratum corneum surface and approaches a more neutral pH below this barrier (6–9). To survive in this environment, bacteria have developed several acid tolerance mechanisms that fall into two main categories: efflux of protons to the extracellular environment and generation of acid-neutralizing compounds (10). For example, the generation of the acid-neutralizing compound ammonia from arginine or urea is a common mechanism found in several bacterial pathogens, including *Streptococcus* sp. and *Helicobacter pylori*, respectively (11). The arginine deiminase pathway, composed of enzymes ArcA (E.C. 3.5.3.6), ArcB (E.C. 2.1.3.3), and ArcC (E.C. 2.7.2.2), is highly conserved in bacteria and contributes to pH homeostasis in *Staphylococcus epidermidis* biofilms (12). Community-associated *S. aureus* and *S. epidermidis* strains often encode a second copy of these genes as part of the arginine catabolism mobile element (ACME) operon, which has been shown to promote *S. aureus* growth in acidic media containing lactic acid and arginine (13). Urease (E.C. 3.5.1.5) is a Ni(II)-dependent enzyme that catalyzes the hydrolysis of urea into ammonia and carbamate, the latter of which spontaneously decomposes into ammonia and bicarbonate (14). The role of urease in pathogenesis is best characterized in *H. pylori*, and it has been shown to promote persistence of *Staphylococcus saprophyticus* in the urinary tract and *S. aureus* in the kidneys (15, 16). However, the role of urease in pH homeostasis during *S. aureus* skin colonization has not been defined.

The concentration of urea in human sweat is reported between 4 and 12 mM, which is produced by ubiquitously distributed eccrine glands on human skin (17, 18). Mice, a common animal model for the study of skin colonization and infections, only have eccrine glands on their paws and ears, and these glands are not used for thermoregulation as in humans (19). Additionally, recent work has demonstrated that the mouse skin surface is neutral (20), which produces challenges in finding appropriate models for studying *S. aureus* acid tolerance on the skin. Recently, development of a skin-like media (SLM) has made it possible to study the role of *S. aureus* urease in a skin-like context (21). This media contains relative concentrations of amino acids representative of the moisturizing factor, as well as metabolites derived from human eccrine glands (17, 22). Additionally, prior transcriptional analysis of *S. aureus* grown in SLM and colonizing mouse skin found that the urease operon is upregulated compared to the control, suggesting a role for urease in *S. aureus* colonization of human skin (21, 23).

Using SLM, we investigated the role of urease in *S. aureus* pH homeostasis on the skin surface during transient skin colonization. We found that a urease-null *S. aureus* strain had reduced growth yield after 24 hours of growth in SLM and observed that urease contributes to intracellular and extracellular pH homeostasis. We determined that SAUSA300_2237, a putative urea transporter of vertebrate origin encoded in *S. aureus*, was necessary for optimal production of extracellular ammonium, suggesting that it facilitates transport of urea. We also determined that the urease inhibitor fluorofamide effectively inhibited *S. aureus* urease activity for at least 24 hours at micromolar concentrations. Together, these data suggest that urease contributes to *S. aureus* pH homeostasis in the skin surface environment and identifies a method of effective urease inhibition.

## RESULTS

### Urease is upregulated in laboratory skin models

**Fig 1:**
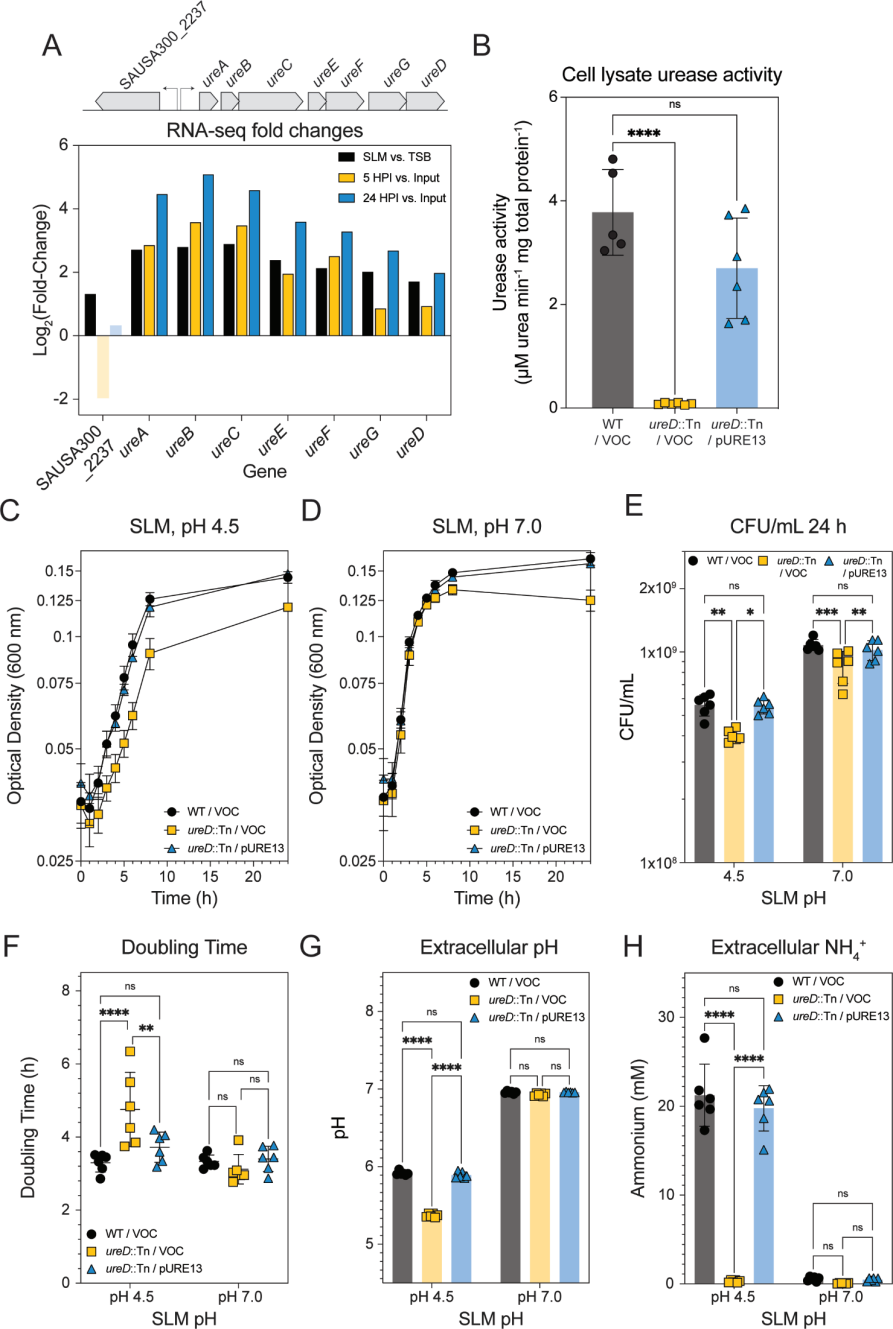
Urease contributes to *S. aureus* growth in skin-like conditions. (**A**) Gene organization of the putative urea transporter and urease operon in MRSA strain LAC is shown above the log_2_ fold changes of these genes in three comparisons from two previously published RNA-seq data sets. Log_2_ fold changes with a *p_adj_* > 0.05 are faded out. (**B**) Urease activity in cell lysates of each strain following 24 hours of growth in SLM. (**C**) Growth of parent strain (WT/VOC), *ureD* mutant (*ureD*::Tn/VOC), and complementation strain (*ureD*::Tn/pURE13) in SLM pH 4.5. (**D**) Growth of parent strain (WT/VOC), *ureD* mutant (*ureD*::Tn/VOC), and complementation strain (*ureD*::Tn/pURE13) in SLM adjusted to pH 7.0. (**E**) Differences in optical density shown in (**B, C**) were confirmed by enumerating CFU/mL. (**F**) Doubling time for each biological replicate, calculated using the exponential (Malthusian) growth equation in Graphpad Prism. (**G**) Extracellular pH of the growth curve shown in (**B–C**) after 24 hours using the methyl red assay for SLM pH 4.5 and the phenol red assay for SLM pH 7.0. (**F**) Extracellular ammonium concentration of the growth curve shown in (**B, C**) after 24 hours. Data shown in (**B–H**) were from two experiments, each with three biological replicates. Statistics in (**B**) were calculated with a one-way ANOVA with a post-hoc Tukey’s test. (**E–H**) were calculated with a two-way ANOVA with a post-hoc Tukey’s test. *P < .05, **P < 0.01, ***P < 0.001, ****P < 0.0001.

Two RNA-seq data sets collected in laboratory skin models were analyzed for transcriptional changes in functions contributing to urea metabolism. The first data set was collected from *S. aureus* strain LAC grown in skin-like media (SLM) and compared to a culture grown to the same density in tryptic soy broth (TSB) (21). The second data set was collected from healthy mouse skin colonized with *S. aureus* strain LAC for 5 and 24 hours and compared to an input culture (23). Analysis of these data sets showed that the genes encoding the enzyme urease (SAUSA300_2238-SAUSA300-2244, *ureABCEFGD*) were significantly upregulated in both data sets compared to the respective reference (Fig. 1A). The putative urea transporter encoded upstream of the urease operon (SAUSA300_2237, *ureT*) was upregulated in SLM compared to TSB but not significantly changed in the mouse skin data set at either 5 or 24 HPI compared to input (Fig. 1A). In summary, these transcriptional data sets show that *S. aureus* upregulates urease transcription in laboratory skin models, suggesting that urease may play a role in pH homeostasis on the skin.

### Urease contributes to optimal growth in SLM

Following the observations from the RNA-seq experiments, we proceeded to investigate whether urease contributes to *S. aureus* growth in human skin-like conditions. We generated a urease-null mutant by moving the *ureD::*Tn mutant from the Nebraska Transposon Mutant Library into our LAC parent background (24). We also complemented this mutant with a plasmid expressing a copy of *ureD* under the control of a constitutive promoter (25, 26) (Table 1). This strain construction approach proved to be more suitable than a clean knockout of the urease operon (Δ*ureABCEFGD,*Δ*ure*), which resulted in ~150-fold upregulation of SAUSA300_2237, a putative urea transporter that was not complementable by insertion of the complete *ureABCEFGD* operon at the φ11 attachment site (Δ*ure*φ11::URE2) (Fig. S1). We confirmed the loss of urease activity in the mutant strain and reconstitution of activity in the complementation strain using a coupled enzyme assay of the cell lysates (Fig. 1B).

**TABLE 1:** Strain information^*a*^

Strain number	Species/strain background	Genotype	Reference
AH1263	*S. aureus* LAC*	WT	(27)
AH4454	*S. aureus* LAC*	WT / pCM28 (VOC)	(28)
AH5871	*S. aureus* LAC*	φ11::LL29erm (VOC)	This work
AH5874	*S. aureus* LAC*	*∆ureABCEFGD*φ11::LL29erm (VOC)	This work
AH5875	*S. aureus* LAC*	*∆ureABCEFGD*φ11::URE2	This work
AH6289	*S. aureus* LAC*	*∆ureT*φ11::LL29erm (VOC)	This work
AH6290	*S. aureus* LAC*	*∆ureT*φ11::URE4	This work
AH6449	*S. aureus* LAC*	*ureD*::Tn / pCM28 (VOC)	This work
AH6450	*S. aureus* LAC*	*ureD*::Tn / pURE13	This work
AH6492	*S. aureus* LAC*	*amt::*Tn	This work (24)
AH6516	*S. aureus* LAC*	*atl::*Tn(tet) / pCM28 (VOC)	This work (24)
AH6517	*S. aureus* LAC*	*atl::*Tn(tet) *ureD::*Tn / pCM28 (VOC)	This work (24)
AH6518	*S. aureus* LAC*	*atl::*Tn(tet) *ureD::*Tn / pURE13	This work (24)
AH6677	*S. aureus* LAC*	WT / pCG44	This work (29)
AH6678	*S. aureus* LAC*	*ureD*::Tn / pCG44	This work (29)
AH5786	*E. coli* DC10B	WT / pURE1	This work
AH6283	*E. coli* DC10B	WT / pURE2	This work
AH6211	*E. coli* DC10B	WT / pURE3	This work
AH6287	*E. coli* DC10B	WT / pURE4	This work
AH6697	*E. coli* DC10B	WT / pURE13	This work

^*a*^ Strain background, genotype, and reference work where applicable for each strain used in this study. VOC = vector-only control. LAC* = *Staphylococcus aureus *strain LAC cured of plasmid pUSA03 that confers erythromycin resistance.

The wild-type (WT) strain (LAC/VOC, vector-only control), the urease-null mutant (LAC *ureD*::Tn/VOC), and urease complement strain (LAC *ureD*::Tn/pURE13) were grown in SLM for 24 hours (Fig. 1C). We also grew these same strains in SLM buffered at pH 7.0 as described previously (Fig. 1D) (21). At 24 hours in both acidic and neutral conditions, there was a significant reduction in colony-forming units (CFU) in the urease mutant that was rescued by complementation (Fig. 1E). Calculation of the doubling time between two and six hours in SLM at pH 4.5, and two and six hours in SLM at pH 7, demonstrated a significant increase in the doubling time of the urease mutant only at pH 4.5 (Fig. 1F). Previous work showed that urea metabolism by urease results in an increased extracellular pH, presumably due to the protonation of the ammonia generated (30). Analysis of culture supernatants grown in SLM at pH 4.5 and pH 7.0 for 24 hours supports these previous observations. In SLM at pH 4.5, there is a significant reduction in extracellular pH for the urease mutant compared to the WT and complement strains, and there was no difference in the extracellular pH with any of the strains grown at pH 7.0 (Fig. 1G). We also measured extracellular ammonia generation under these conditions and found that in SLM at pH 4.5, there was a significant production of extracellular ammonia in the WT and complementation strain, which was not observed in the urease mutant (Fig. 1H). Interestingly, in SLM at pH 7.0, we did not observe significant production of extracellular ammonia, suggesting that this is a pH-dependent phenotype. These results support a role for urease in contributing to growth and extracellular pH homeostasis in the acidic skin environment.

### Urease activity contributes to intracellular pH homeostasis in *S. aureus*

**Fig 2:**
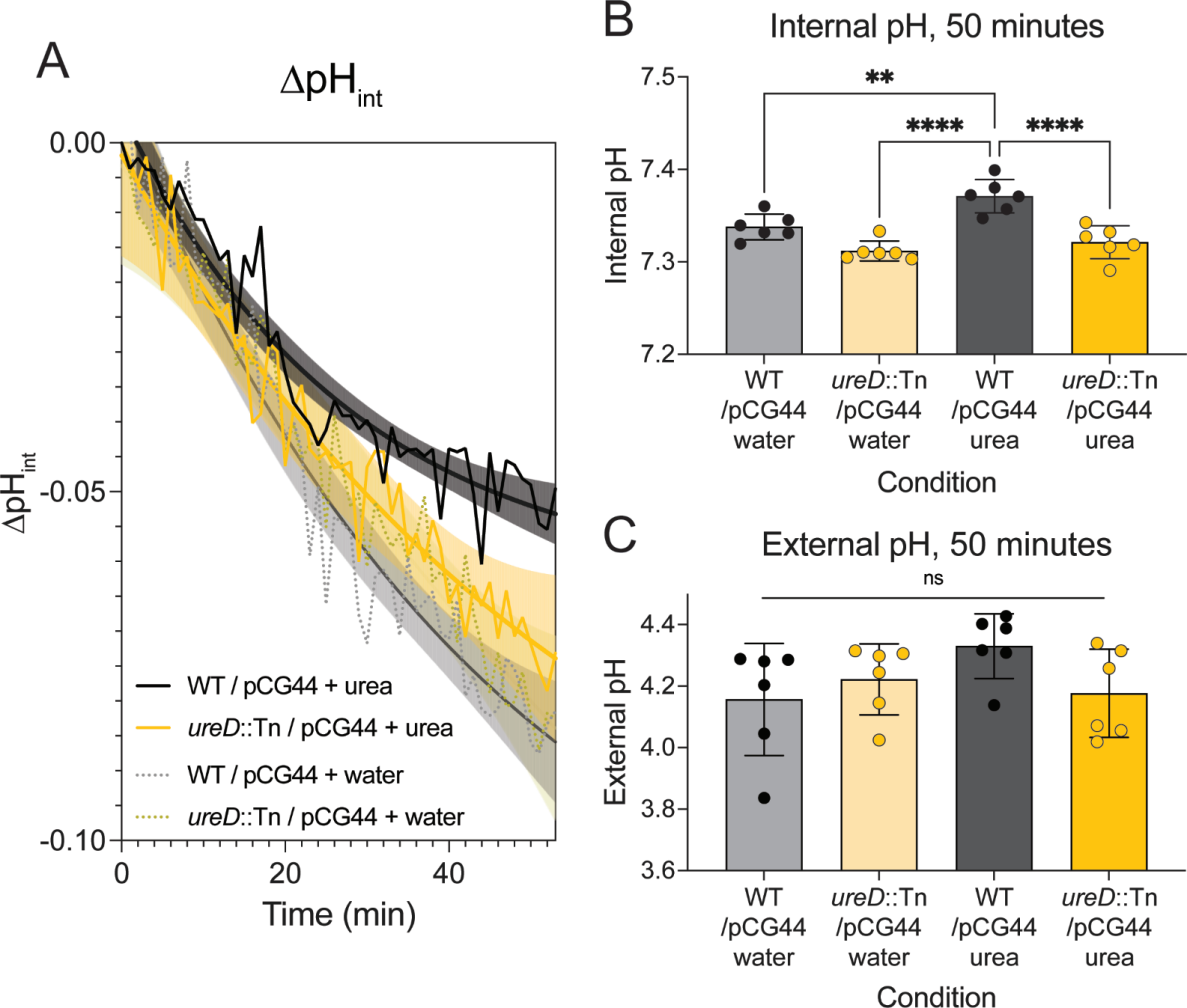
Urea metabolism contributes to intracellular pH homeostasis. WT (black) and *ureD*::Tn (yellow) strains transformed with plasmid pCG44 encoding a pH-sensitive GFP, pHluorin, were grown in biological triplicate and assessed for changes in intracellular pH for 50 minutes after addition of urea or an equivalent volume of water. (**A**) A plot of the change in intracellular pH over time post-substrate addition. WT + urea (black solid line), *ureD::*Tn + urea (yellow solid line), WT + water (gray dotted line), or *ureD::*Tn + water (dark yellow dotted line). Each strain was fitted to a one-phase decay curve, with the shaded region indicating the 95% CI of the curve. (**B**) A bar plot of the intracellular pH at the last time point. (**C**) Immediately after the last time point, the cultures were centrifuged, and the supernatant pH was measured for each sample using the methyl red assay. Data are from two experiments, with three biological replicates for each experiment. For (**B**) and (**C**), statistics were calculated using one-way ANOVA with a post-hoc Tukey test. **P < 0.01, ****P < 0.0001.

The observation that the extracellular pH was modified led us to investigate whether urease, which is an intracellular enzyme, was also contributing to intracellular pH. We transformed LAC WT and *ureD::*Tn strains with plasmid pCG44, which encodes a pH-sensitive fluorescent reporter used to measure intracellular pH (31). Cultures were grown in TSB overnight in biological triplicate, washed with water, and diluted into a buffer that was comprised of the salt and mineral components of SLM. These cultures were monitored by fluorescence, and a solution of urea or an equivalent volume of water was spiked in, followed by 50 minutes of continuous monitoring. As shown in Fig. 2A, the change in intracellular pH of WT/pCG44 with urea added begins to deviate at around 20 minutes compared to WTpCG44 with water added or *ureD::*Tn/pCG44 with either urea or water added. When the intracellular pH of all samples was compared at 50 minutes post-substrate addition, the intracellular pH of WT + urea was significantly higher than all other conditions tested (Fig. 2B). At this time point, the samples were also centrifuged, and the culture supernatant pH was measured using the methyl red assay. No significant differences were found in the extracellular pH, suggesting that urease is directly contributing to increasing the intracellular pH as well as eventually increasing the extracellular pH (Fig. 2C).

### Urea metabolism is a shared good

**Fig 3:**
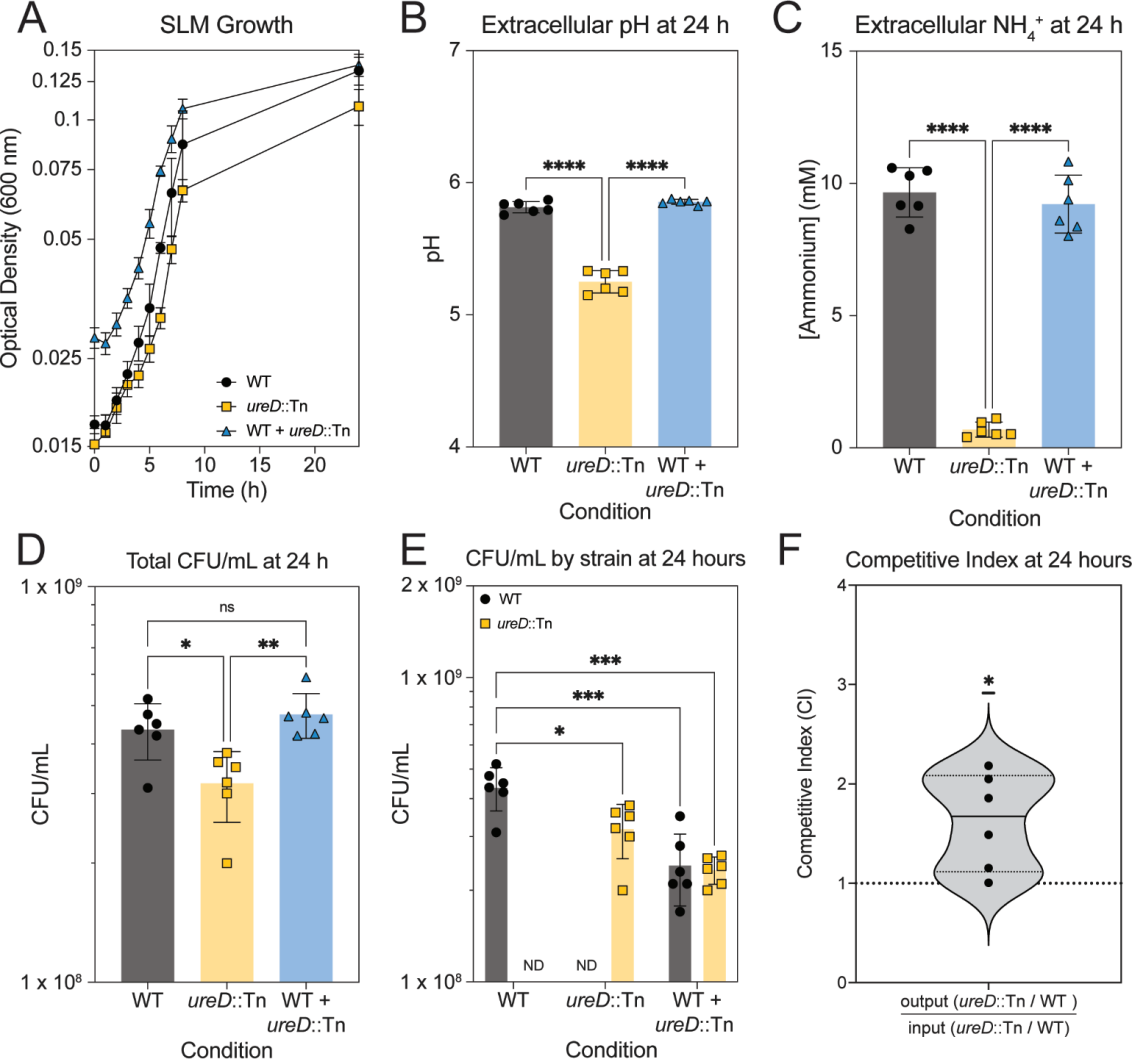
Urease activity benefits urease-deficient strains in coculture. (**A**) Growth curve of WT (black circles), *ureD*::Tn (yellow squares), and WT and *ureD::*Tn mixed together (blue triangles) in SLM over 24 hours. (**B**) Extracellular pH at 24 hours. (**C**) Extracellular ammonium concentration at 24 hours. (**D**) Total CFU/mL of each condition as measured by dilution plating on nonselective media. (**E**) CFU/mL of each strain in each condition as determined by selective plating. ND = not detected. (**F**) Competitive index of urease activity as calculated by dividing the CFU/mL of WT and *ureD*::Tn in the coculture condition. Statistics for **B–D** were calculated using a one-way ANOVA with post-hoc Tukey test. Statistics for **E** were calculated using a two-way ANOVA with a post-hoc Tukey test. Statistics for F were calculated using a one-sample Wilcoxon t test. ns = not significant. *P < 0.05, **P < 0.01, ***P < 0.001, ****P < 0.0001. ND = not detected.

Since urea metabolism is contributing to both intracellular and extracellular pH, we wanted to test whether urea metabolism could be considered a “shared good”—that is, does metabolism of urea by one cell also benefit an adjacent urease-deficient cell? Shared, or public, goods in a microbial population are often regulated by quorum sensing, and it has been shown previously that the urease operon is regulated by the accessory gene regulator Agr (32). We grew WT and *ureD*::Tn alone and in coculture in SLM for 24 hours to assess whether urea metabolism provides a competitive advantage to WT. At 24 hours, we observed no difference in growth (Fig. 3A), extracellular pH (Fig. 3B), ammonium generation (Fig. 3C), or total CFU/mL (Fig. 3D) in the coculture compared to the WT strain alone, whereas we did observe a significant reduction in these parameters in the *ureD*::Tn monoculture. The cultures were also dilution plated on plates selective for the transposon mutant to calculate the fraction of WT and *ureD::*Tn in the co-culture. We found that the CFU/mL was significantly lower for *ureD::Tn* monoculture compared to WT monoculture but was not significantly different in the coculture or in the input culture (Fig. 3E; Fig. S2). We calculated the competitive index score using the equation $CI=\frac{outputmutant÷outputWT}{inputmutant÷inputWT}$, and found a mean competitive index of 1.623 ± 0.4841 standard deviation, suggesting that urea metabolism by WT slightly benefits *ureD::*Tn growth in SLM (Fig. 3F).

### The urease-dependent growth defect is only rescued by ammonium at neutral pH.

**Fig 4:**
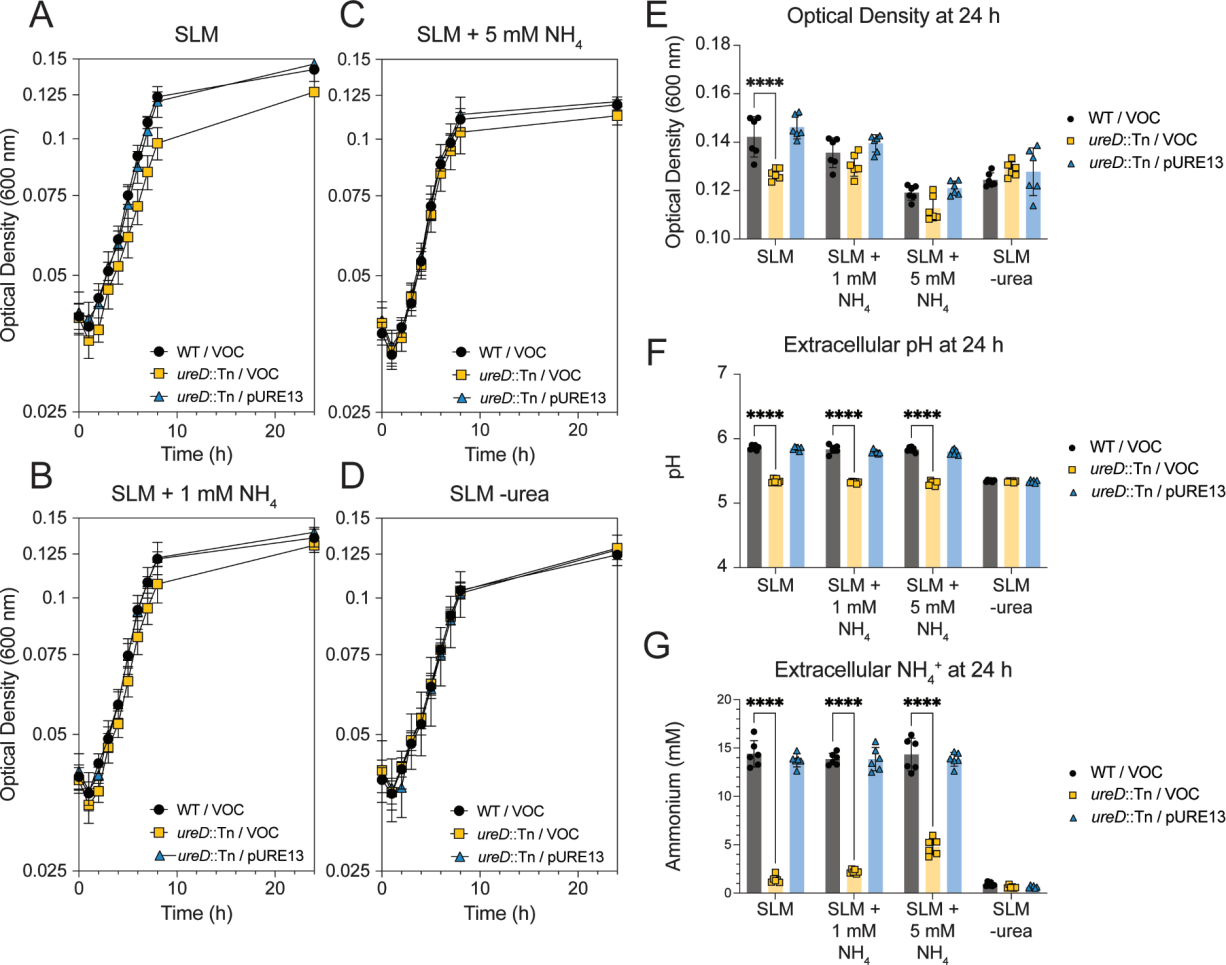
The *ureD*::Tn growth defect cannot be complemented by the addition of ammonium. Growth of WT/VOC (black circles), *ureD*::Tn/VOC (yellow squares), and *ureD*::Tn/pURE13 (blue triangles) in (**A**) SLM, (**B**) SLM + 1 mM NH_4_, (**C**) SLM + 5 mM NH_4_, and (**D**) SLM -urea. (**E**) A bar graph of culture absorbance at 24 hours from growth curves in (**A–D**). (**F**) A bar graph of culture supernatant pH at 24 hours from growth curves in (**A–D**). (**G**) A bar graph of ammonium concentration in culture supernatant at 24 hours from growth curves in (**A–D**). Data shown are from two experiments, each experiment performed in biological triplicate. Statistics were calculated using two-way ANOVA, with a post-hoc Dunnett’s test. ****P < .0001.

The SLM recipe contains 10 mM urea and a total of 1.25 mM of amino acids as potential nitrogen sources. Because we observed a growth defect with the *ureD*::Tn mutant at 24 hours in SLM at both acidic and neutral pH, it is possible that the cells are experiencing nitrogen limitation. To determine if the urease-dependent growth defect is due to nitrogen limitation, SLM was supplemented with 1 mM or 5 mM ammonium. Additionally, SLM lacking urea was tested. We also repeated these experiments in SLM at pH 7.0. Addition of ammonium at 1 mM reduced optical density of WT and complement strains in SLM and did not improve growth of the urease-null strain (Fig. 4A through E; Fig. S3). The addition of ammonium at 5 mM significantly reduced optical density of all three strains (Fig. 4E; Fig. S3D). As expected, the addition of ammonium did not contribute to urease-dependent increases in extracellular pH (Fig. 4F) or extracellular ammonium (Fig. 4G). When these strains were grown in SLM at pH 7.0, addition of 5 mM ammonium significantly improved growth of the *ureD*::Tn mutant at 24 hours (Fig. S4A through E), but not at earlier time points. There were also slight but significant differences in the extracellular pH that correlated to generation of a small amount of extracellular ammonia that was urease-dependent (Fig. S4F and G). The data collected from SLM at neutral pH suggest that at late log and stationary phases of growth, *S. aureus* does experience some nitrogen limitation which can be corrected by supplementation with ammonium. Conversely, at acidic pH, supplementation of ammonium to correct nitrogen limitation inhibits growth.

### Amino acid supplementation does not phenocopy urea supplementation

**Fig 5:**
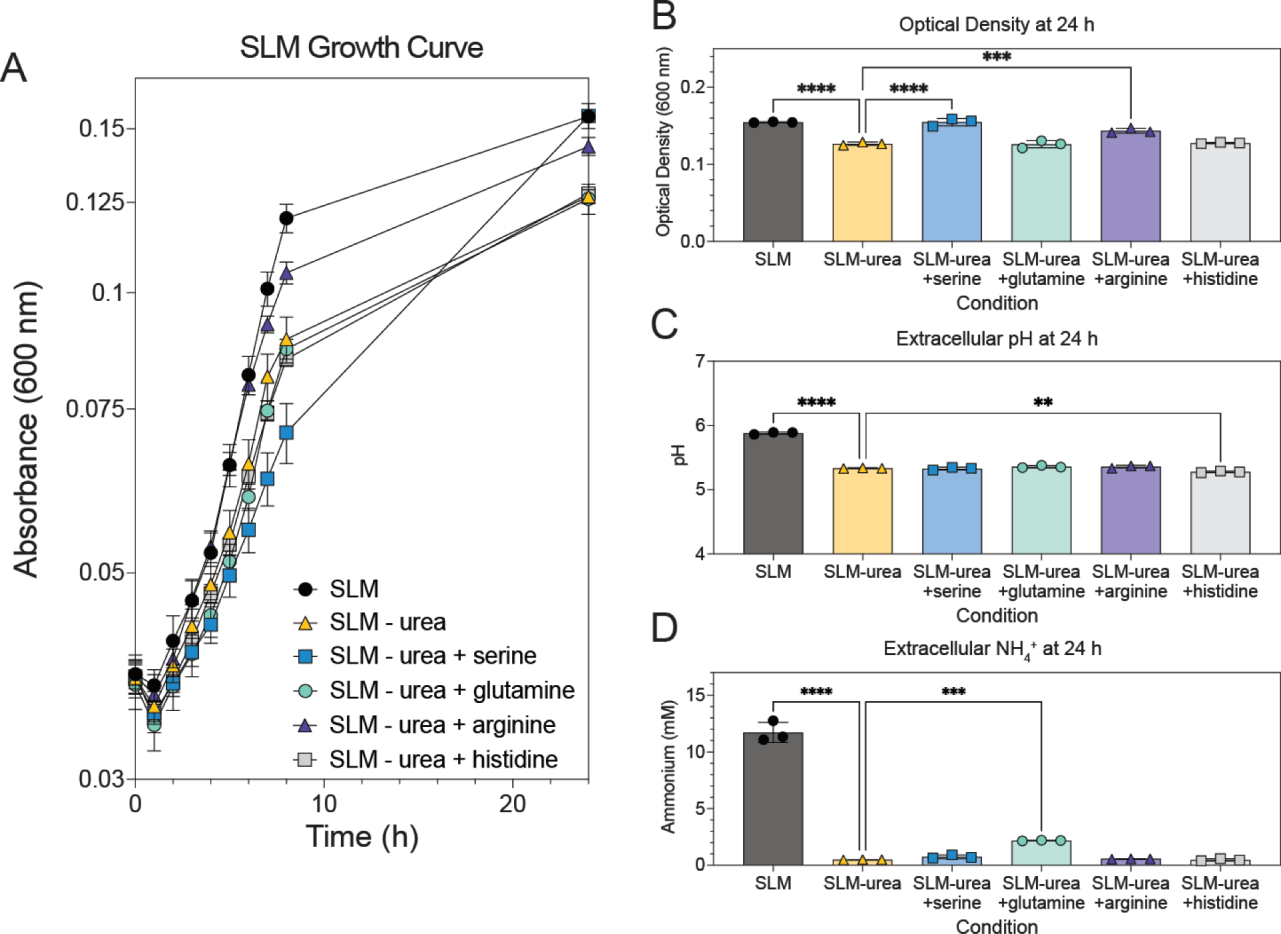
Addition of alternative ammonia generators does not fully complement urea metabolism phenotype. (**A**) WT/VOC strain was grown in SLM (black circles), SLM – urea (white triangles), SLM – urea + serine (yellow squares), SLM – urea + glutamine (blue circles), SLM – urea + arginine (teal triangles), and SLM – urea + histidine (purple squares). (**B**) Addition of urea, serine, or arginine to SLM – urea improved growth significantly. (**C**) Addition of urea to SLM – urea significantly increased the extracellular pH, whereas addition of histidine significantly reduced extracellular pH. (**D**) Urea and glutamine significantly increased the concentration of extracellular ammonia. Data are from one experiment, performed in biological triplicate. Statistical analysis was performed with a one-way ANOVA, with a post-hoc Dunnett’s test comparing to SLM – urea. **P < 0.01, ***P < 0.001, ****P < 0.0001.

Amino acids, including arginine, are known sources of ammonia which also contribute to pH homeostasis. The concentrations of these amino acids are low in SLM, and to assess if they can replace urea in exogenous ammonia generation and extracellular pH, urea was omitted from SLM and supplemented with 10 mM serine, glutamine, arginine, or histidine. After 24 hours of incubation, supplementation of arginine improved optical density of LAC WT, corroborating previous observations (Fig. 5A) (13). Serine also significantly improved optical density of LAC WT at 24 hours, although it had a slower growth rate (Fig. 5A). The optical density improvement observed with serine and arginine supplementation was not accompanied by an increase in extracellular pH (Fig. 5B) or extracellular ammonium (Fig. 5C), as was observed with addition of urea. In the absence of urea, the urease mutant largely phenocopied the WT strain, demonstrating that the urease mutant does not influence utilization of these amino acids (Fig. S5). These results suggest that the mechanism with which urease contributes to pH homeostasis is different from that of deamination of amino acids such as arginine, and the ammonium generated from urea is largely directed to the extracellular environment as opposed to being incorporated into central metabolism (13).

### Cell lysis does not contribute to extracellular ammonium generation

Since urease is an intracellular enzyme, it was unclear how the ammonia generated from urea metabolism is transported to the extracellular environment. There is some evidence in the literature that in *Helicobacter pylori*, extracellular urease originating from a small group of lysed cells in the population contributes to extracellular ammonia generation (33). However, the relevance of this model in infection has been debated (34). In *S. aureus*, the major cell wall hydrolase autolysin A plays a critical role in cell lysis, and thus, we hypothesized that if urease is being released to the extracellular environment through lysis, an autolysin mutant would reduce this release. To explore this possibility, we washed overnight cultures of WT and autolysin A mutant (∆*atl*) strains to remove extracellular debris. Half of the WT culture was then incubated with lysostaphin for 20 minutes as a positive lysis control. These cultures were subcultured in SLM over an eight-hour period, and at several time points, filtered supernatant was collected to assess extracellular pH and ammonium concentration (Fig. S6). There were no differences in optical density (Fig. S6A), extracellular pH (Fig. S6B), or extracellular ammonia generation (Fig. S6C) between WT and ∆*atl* after eight hours. The partially lysed culture started at a lower culture density as expected, and the contents of the lysed cells did not result in comparable or higher extracellular pH or extracellular ammonium generation compared to WT. These results suggest that extracellular urease does not significantly contribute to extracellular ammonium generation in these conditions.

### Role of the putative urea transporter on extracellular ammonium generation

**Fig 6:**
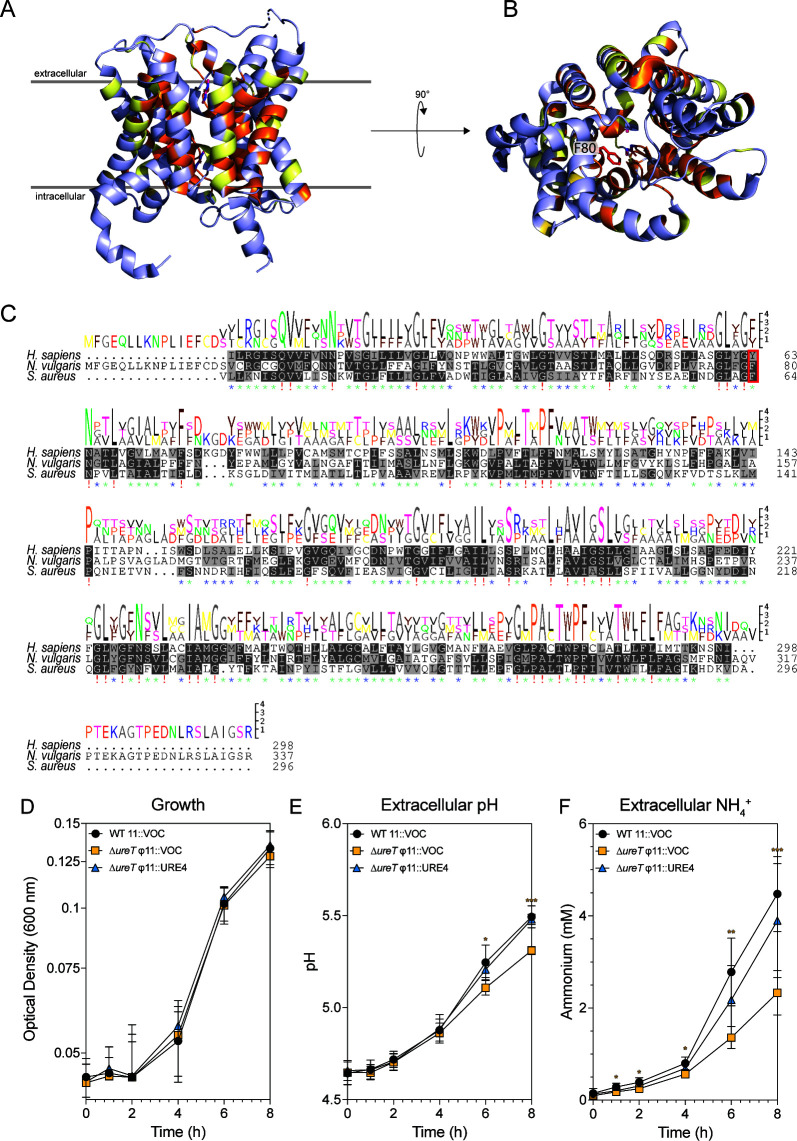
The putative urea transporter SAUSA300_2237 (*ureT*) facilitates extracellular ammonia generation. (**A**) Side view and (**B**) bottom-up view of the crystal structure of UreT (PDB: 3K3F) from *Nitrodesulfovibrio vulgaris* (*Nv*UreT). Similar residues between *Nv*UreT, *Homo sapiens* urea transporter, and *S. aureus* UreT are shown in yellow; identical residues are shown in orange. (**C**) A multiple sequence alignment showing amino acid conservation across the three UreT homologs analyzed. Amino acid conservation is shown above the alignment. Amino acid identity is indicated below the alignment. 100% amino acid identity = red exclamation mark, >60% amino acid identity = green asterisk, >60% amino acid similarity = blue asterisk. (**D**) Growth curve of cultures grown for eight hours in SLM. (**E**) Measurement of extracellular pH over eight hours of growth in SLM. (**F**) Measurement of extracellular ammonium over eight hours of growth in SLM. Data shown in (**D–F**) are from two experiments, each performed in biological triplicate. Statistical analysis was performed by two-way ANOVA with the Geisser-Greenhouse correction, with matched values stacked into a subcolumn. Post-hoc Dunnett’s test was performed with individual variances computed for each subcolumn. *P < 0.05, **P < 0.01, ***P < 0.001.

*S. aureus* LAC encodes a putative urea transporter (SAUSA300_2237, *ureT*) upstream of the urease operon. This transporter shares 26% homology with the human urea transporter 1, isoform 2 (locus WRX36614), and 30% homology with a structurally and functionally characterized urea transporter from *Nitrodesulfovibrio vulgaris*, formerly *Desulfovibrio vulgaris* (locus WP_010938457, PDB 3K3G) (Fig. 6A and B) (35). UreI, the urea transporter in *Helicobacter pylori*, does not share detectable amino acid sequence similarity with these transporters using NCBI protein BLAST with standard parameters. In *N. vulgaris*, the transporter facilitated uptake of radiolabeled urea at a faster rate compared to diffusion of urea across the lipid membrane (35). A multiple sequence alignment between *S. aureus ureT*, *H. sapiens* urea transporter 1, and *N. vulgaris ureT* reveals several conserved residues across the three homologs that align to the core of the transporter structure, including a shared phenylalanine residue (F80) in *S. aureus* UreT and *N. vulgaris* UreT that was critical for urea transport in *N. vulgaris* (Fig. 6B and C) (35). To determine if the putative urea transporter plays a role in urea metabolism and extracellular ammonium generation, a clean deletion of *ureT* was made (∆*ureT* φ11::VOC), and a strain with *ureT* and its native promoter at a chromosomal insertion site (∆*ureT*φ11::URE4) for complementation was generated. These strains and a WT control (φ11::VOC) were grown in SLM, and the growth was measured by absorbance over a period of eight hours (Fig. 6D). At several time points, the culture supernatant was harvested, and the extracellular pH (Fig. 6E) and extracellular ammonium (Fig. 6F) were measured. Although there were no significant differences in growth, a significant difference in extracellular pH and extracellular ammonium generation was observed, suggesting that facilitated urea transport by UreT plays a role in extracellular ammonia generation. After 24 hours in growth, there is a very small difference in extracellular pH and no difference in extracellular ammonium, further supporting the role of UreT in facilitation of urea transport (Fig. S7). Since the deletion of *ureT* did not completely prevent generation of extracellular ammonium, we investigated whether the putative ammonium transporter (SAUSA300_1996, *amt*) was contributing to the transport of ammonium to the extracellular environment. We grew WT and the transposon mutant *amt::*Tn in SLM and found no difference in ammonium concentration in the culture supernatant between these strains after eight hours of growth (Fig. S8A through B).

### Fluorofamide effectively inhibits *S. aureus* urease.

**Fig 7:**
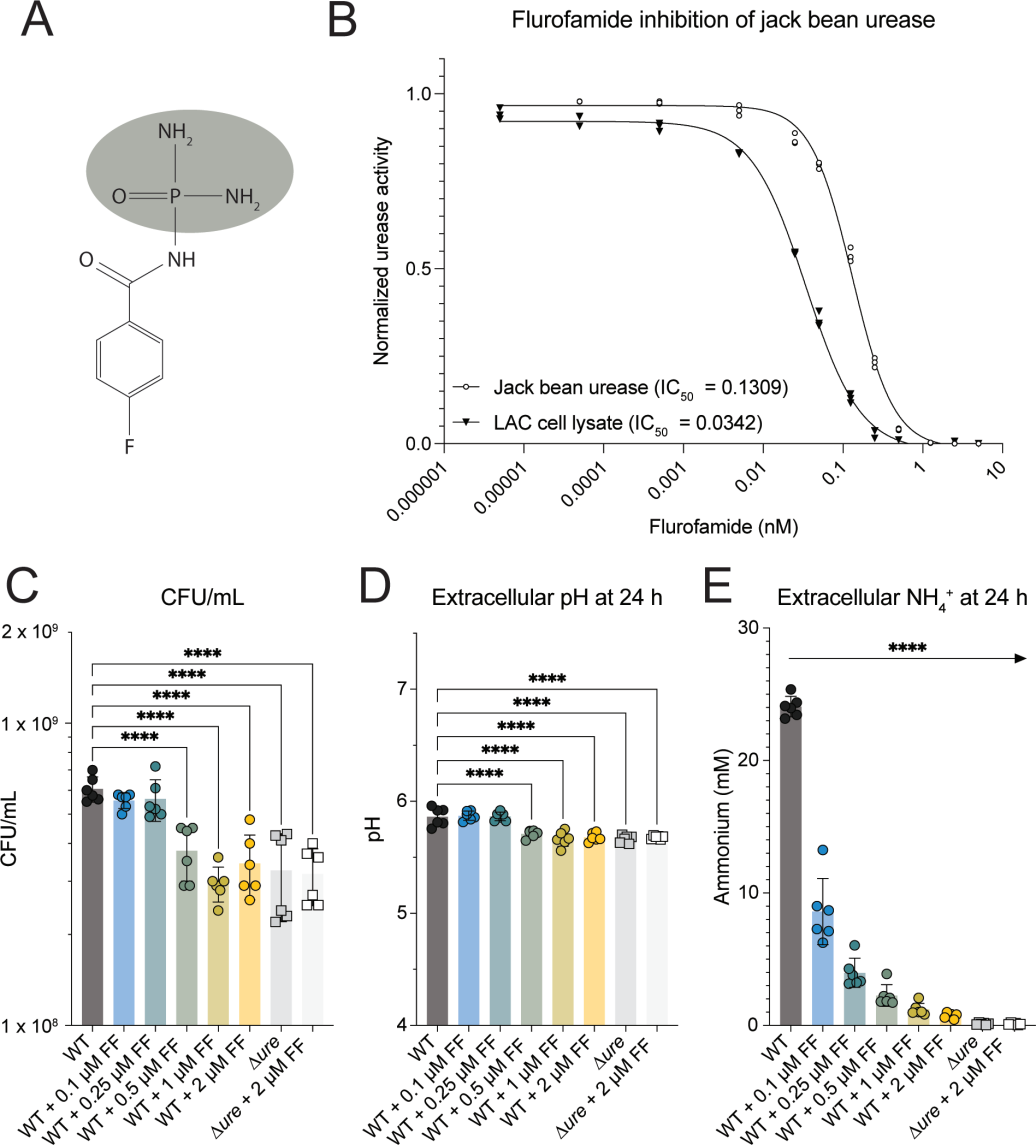
Fluorofamide is a potent inhibitor of *S. aureus* urease activity. (**A**) Chemical structure of fluorofamide (FF), outlined in gray, is the region that mimics urea. (**B**) Inhibition of urease activity in *S. aureus* cell lysate and purified jack bean urease and the calculated IC_50_ of fluorofamide for each. (**C**) Inhibition of growth in SLM by addition of fluorofamide, as determined by colony-forming units per mL. (**D**) Reduction in extracellular pH upon addition of fluorofamide. (**E**) Reduction of extracellular ammonia upon addition of fluorofamide. The curve in (**B**) was fitted using Graphpad’s [Inhibitor] vs. response – variable slope. Data in (**B**) are from one biological experiment, performed in biological triplicate. Data in (**C–E**) are from two experiments, performed in biological triplicate. Statistical analysis in (**C–E**) was calculated with one-way ANOVA with a post-hoc Dunnett’s test comparing to WT with no fluorofamide added. ****P < 0.0001.

Fluorofamide (N-(diaminophosphinyl)-4-fluorobenzenamide, FF, Fig. 7A) is a potent inhibitor of urease. The application of FF was previously shown to be effective at urease inhibition in *Helicobacter pylori*; however, it was only partially effective with related species *Staphylococcus saprophyticus* (36, 37). To assess whether FF could effectively inhibit *S. aureus* urease, cell-free lysates of *S. aureus* LAC were prepared, and urease activity was measured with incubation of increasing concentrations of FF in the presence of 50 mM urea (Fig. 7B). Inhibition of commercially purified jack bean urease was also tested. *S. aureus* urease activity was successfully inhibited by FF, with an IC_50_ = 34.2 nM (Fig. 7B). In comparison, FF was less inhibitory for jack bean urease, which had an IC_50_ = 130.9 nM (Fig. 7B). We next investigated whether FF could inhibit urease activity with intact cells, as FF has been reported to be membrane impermeable (38). We found that colony-forming units and culture supernatant pH were significantly reduced when 0.5 µM FF was added, and extracellular ammonia generation was significantly reduced at 0.1 µM FF addition. The effect of FF on *S. saprophyticus* was reported to be temporary, whereas FF continued to inhibit urease activity for at least 24 hours. These data suggest that FF is a potent inhibitor of *S. aureus* urease.

## DISCUSSION

**Fig 8:**
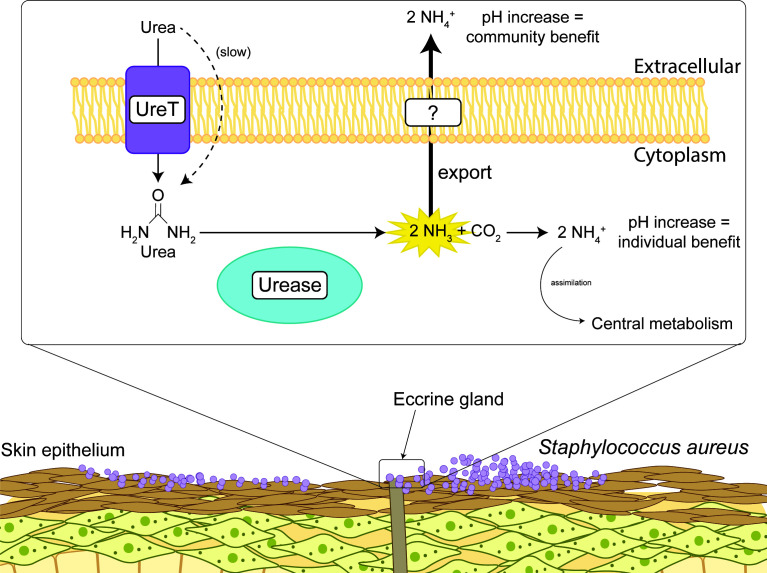
A model of the role of urea metabolism in *S. aureus* pH homeostasis. A diagram depicts the upper epithelial layers of the skin transiently colonized by *S. aureus*. An eccrine gland, which produces sweat containing urea, is also depicted in the figure. A close-up of the cell membrane and cytoplasm of *S. aureus* colonizing the skin is depicted in the rectangular inset at the top of the figure. The rectangular inset depicts urea metabolism in *S. aureus*: urea diffuses through the membrane slowly or is more quickly transported into the cell by UreT, where it is metabolized by Ni(II)-dependent urease into carbon dioxide and ammonia. The ammonia, protonated either intracellularly and exported through an unknown mechanism (denoted by the question mark), or protonated extracellularly, raises the extracellular pH.

In this work, we demonstrate the contribution of urease to *S. aureus* pH homeostasis in the skin environment (Fig. 8). We show in a previously developed *in vitro* model of the human skin surface’s metabolite composition that urease activity contributes to growth yield and increase of both intracellular and extracellular pH (21). We further demonstrate that the role of urease is primarily in pH homeostasis, and urea metabolism, as measured by extracellular ammonia generation, is optimized by an encoded urea transporter. We also show that the urease inhibitor fluorofamide is effective at inhibiting urease activity in *S. aureus* cultures at micromolar concentrations.

Our investigations began with the observation that the urease operon was upregulated in *in vitro* and *in vivo* skin surface-like conditions (Fig. 1). *S. aureus* does not encode a urea-responsive transcriptional regulator, and urease expression is instead regulated by a consortia of regulators, including catabolite control protein A (CcpA), accessory gene regulator (Agr), global regulator CodY, two-component system ArlRS, and regulator MgrA (16, 39, 40). Although urea on mouse skin has not been quantified to date, the lack of eccrine glands excreting urea likely changes the available concentration of this metabolite on murine skin, compared to human skin. Thus, the coupling of urease regulation to urea-independent factors could explain why we see upregulation on murine skin.

Using our *in vitro* model, skin-like media, we observed that our urease-deficient *S. aureus* strain, *ureD*::Tn, demonstrated reduced growth in SLM (Fig. 1). Although this phenotype was significant during the time course performed, it was subtle. A prior study documented *S. aureus* colonization of human skin for a median of 21 days, a time course which is beyond the scope of this and other contemporary skin colonization models (41). Future improvements to the model, including modeling growth in continuous culture, could provide insights into the long-term benefits of urea metabolism and other metabolic pathways for *S. aureus* skin colonization.

The urease-dependent growth defect appeared to be a combination of reduced pH homeostasis at initial growth phases, as evidenced by the change in growth rate observed in SLM at pH 4.5, and nitrogen limitation in the stationary phase, as evidenced by the reduction in culture absorbance in SLM at pH 7.0. When cultures were harvested after 24 hours of growth in SLM, we found that the reduced absorbance at both pH 4.5 and pH 7.0 could be explained by a difference in culture density as confirmed by CFU plating. We also observed that the mutant had reduced extracellular pH and reduced extracellular ammonium after 24 hours of growth in SLM at pH 4.5, and *S. aureus* generated much lower quantities of extracellular ammonia at pH 7.0. These phenotypes were complemented by expression of *ureD* on a plasmid under a constitutive promoter, demonstrating that they were urease-dependent. We calculate that most of the possible ammonia generated from the supplemented urea could be accounted for in the culture supernatant in SLM at pH 4.5. For example, 106 ± 17% in Fig. 1H and 72 ± 6% in Fig. 4 of the possible ammonium generated from urea were measured in the supernatant. In Gram-negatives, such as *Helicobacter pylori*, ammonia generated by urease activity is transported to the periplasm and results in an increase in periplasmic pH (42). In Gram-positives like *S. aureus*, it is possible that urease instead primarily functions to raise the pH of the local extracellular environment.

We found that urease activity contributed to an increase of both intracellular and extracellular pH (Fig. 1–2). The modulation of extracellular pH by urease activity suggests that metabolism of urea could be a “shared good” in a microbial community. Shared or public goods in bacterial communities include production of siderophores, for example, which benefits both producers and non-producers (43). Supporting this hypothesis, we found that the *ureD::*Tn mutant was slightly more competitive in growth to WT in co-culture (Fig. 3). This suggests that urea metabolism benefits the surrounding microbial community by increasing the extracellular pH, which likely promotes an increase in the intracellular pH and mitigation of acid-induced damage. Additionally, the competitive index calculated suggests that urease-deficient “cheaters” may benefit in a mixed community of microorganisms. This observation predicts that urea metabolism will not appear as underrepresented in competition experiments such as transposon sequencing. Curiously, a recently published transposon-sequencing experiment in *S. aureus* identified *ureB* alone as an underrepresented gene in the murine female reproductive tract (44). It has previously been demonstrated that *ureB* in *H. pylori* encodes a small RNA that regulates *ureB* expression (45). The lack of underrepresentation of other urease genes in this transposon-sequencing study would suggest a urea-independent function for the *ureB* locus in *S. aureus* that should be investigated further in future studies.

Due to the phenotypes observed in SLM at neutral pH, there was a possibility that inactivation of urease caused nitrogen starvation in the *ureD::*Tn mutant. We addressed this by adding ammonium to SLM at acidic and neutral pH and found that addition of ammonium slightly improved the growth yield of the *ureD* mutant in SLM at neutral pH and significantly reduced the growth yield of all strains in SLM at acidic pH (Fig. 4; Fig. S4). While the data collected at neutral pH confirm the nitrogen limitation, the reduction in growth yield at acidic pH was puzzling. Data from the literature suggests that it is possible that the supplemented ammonium could impact the proton motive force, which could impact growth of *S. aureus* (46). In addition to ammonium supplementation, we would have expected that addition of any of the supplemented amino acids would have satisfied the nitrogen requirements of the cell and improved growth of the *ureD::*Tn strain in acidic conditions; however, growth improvement was only observed with addition of serine and arginine (Fig. 5) (47). Growth improvement with arginine was previously demonstrated to be attributed to pH homeostasis, and our data support this conclusion (13). It is possible that in acidic conditions, some of these amino acids are not metabolized sufficiently to complement the nitrogen limitation observed or have other effects that result in the lack of a growth benefit. Additional experiments measuring the transport of these metabolites at different external pHs, and the effect of their addition on membrane potential, would be required to better understand the phenotype of *S. aureus* in these growth assays.

Although urea can move through the membrane in acidic conditions, the ammonia generated should protonate in the cytoplasm and would then require facilitated export. In this work, we were not able to identify a transporter responsible for the export of ammonium to the extracellular matrix. We were able to rule out a role for extracellular urease activity due to cell lysis, as well as a role for the putative ammonium transporter Amt (Fig. S3 and S4). Amt has 51% identity to NrgA in *Bacillus subtilis*, which is required for ammonium utilization as a sole nitrogen source in acidic conditions (48). We did find that mutation of the putative urea transporter, UreT, reduced the amount of ammonium found in the culture supernatant (Fig. 6). Whether this is because of slowed urea transport—and therefore slowed urea metabolism—or because the transporter is capable of transporting both urea and ammonium cannot be determined by the available data. Investigations into the urea transporter UreI from *H. pylori* offer a potential hypothesis, as UreI has been demonstrated to interact with urease subunits UreA, UreB, and UreE, and to transport urea, ammonia, and ammonium (42, 49). Although UreT is a larger protein and does not appear to share sequence similarity with UreI, it is possible that UreT is also interacting with urease and facilitating transport of both ammonia and urea. This model could explain the why urea-generated ammonia is transported to the extracellular environment, a phenomenon which is not observed when ammonia-generating amino acids are supplemented to the media in excess (Fig. 5 and 6). However, a problem with this model is that we have shown that mutation of UreT does not completely abrogate generation of extracellular ammonium, and thus, ammonium export must occur through an as-yet unidentified mechanism.

To date, the efficacy of urease inhibitors, such as fluorofamide, has not been studied in *S. aureus*. The closest species studied was *S. saprophyticus*, and the authors of this work observed a temporary and incomplete inhibition of urease activity (36). Here, we show that fluorofamide successfully inhibits *S. aureus* urease activity in micromolar quantities for up to 24 hours and significantly reduces viable cell counts, extracellular pH, and extracellular ammonium generation to levels observed in a urease-deficient strain. It was surprising that fluorofamide inhibited intracellular urease activity, as the molecule is thought to be membrane impermeable. It was previously speculated that fluorofamide is transported by one of the many transporters in *S. saprophyticus*, and we believe this may also be possible for *S. aureus* (36). These results offer evidence for successful inhibition *of S. aureus* urease activity on the skin through topical application of urease inhibitors.

Together, these results offer evidence for the contribution of urease to *S. aureus* pH homeostasis and nutrient acquisition in a skin-like environment, identify a functional role for UreT, and explore the therapeutic potential of urease inhibition by the inhibitor fluorofamide. The effect of urease-deficient strains in an *in vivo* model remains to be demonstrated; however, this is complicated by physiological differences between human skin and current laboratory models. For example, human skin contains ubiquitous sweat glands that are responsible for the concentration of urea found on human skin, and the pH of the human skin surface is lower than that of mice and other animal models (6, 17, 20). With future improvements in *in vivo* and *ex vivo* skin models that better approximate the human skin environment, particularly in relation to pH and sweat gland metabolites, investigations should be undertaken to explore the role of urease in *S. aureus* skin colonization.

## MATERIALS AND METHODS

### Growth media

Tryptic Soy Broth (TSB) was made according to the manufacturer’s instructions and autoclaved before use (RPI Cat. No. T48500). Skin-like media (SLM) at pH 4.5 and pH 7.0 were formulated as previously described (21). Amino acid supplementations were added to SLM as a dry powder to the final media formulation, pH adjusted to match the pH of pre-supplemented SLM, and filter sterilized using a 0.2 µm MCE filter (Millipore Sigma, Cat. No. SLGSR33SS). Antibiotics erythromycin (5 µg/mL) and lincomycin (5 µg/mL) were used where indicated.

### Strain construction

Transposon mutants *ureD*::Tn and *amt::*Tn were obtained from the Nebraska Transposon Mutant Library (24). Transposon insertions were moved into the parent strain background using generalized transducing phage φ11, and successful transductants were PCR verified. The autolysin deletion mutant (∆*atl*) was phenotypically validated using a previously published autolysin assay (50). The ∆*ureT* strain was constructed as described in Table S1 using a previously published homologous recombination protocol and sequence verified (51). The *ureD::*Tn complementation vector (pURE13) and the ∆*ureT* complementation construct (pURE4) were constructed as described in Table S1.

### SLM growth curves

Cultures were grown for 16 hours in 2 mL of TSB in 18 × 150 mm glass tubes, shaking at 250 RPM at 37°C, in biological triplicate. These cultures were diluted 1:100 in 150 µL of SLM in a 96-well plate, in technical duplicate or triplicate, and grown shaking at 1000 RPM at 32°C. Time points were taken with a plate reader (Tecan Infinite Mplex). At desired time points, technical replicates were combined, and supernatants were filter sterilized for pH and ammonium assays (Costar Spin-X Cat. No. 8160). For CFU plating at 24 hours, technical replicates were combined, and the sample was vortexed for 10 seconds before diluting in phosphate-buffered saline (Fisher Cat. No. BP2944), and 10 µL of each dilution were drip-plated onto TSA plates. Plates were incubated overnight at 37°C, and colony-forming units were counted. For assessment of cell lysis, overnight cultures were centrifuged at 8,000 *× g* and washed with saline thrice before reconstituting in fresh TSB. As a positive lysis control, 500 µL of the washed WT culture was partially lysed with addition of 1 µL of lysozyme solution (10 mg/mL = 400 U/mL in 20 mM sodium acetate pH 4.5) for 20 minutes before diluting into SLM for the growth curves. For competition experiments, overnight cultures were normalized to an optical density of 7 and diluted with an equal volume of TSB or mixed the two cultures together 50/50 before diluting into SLM at a 1:100 dilution. Cultures were drip-plated onto TSA or TSA + erythromycin + lincomycin for CFU enumeration.

### Extracellular pH assays

A methyl red solution was generated immediately before the assay by diluting the methyl red stock (1.2 mg/mL in 100% EtOH) 1:100 in Ultrapure water to generate a 5X solution. Eighty microliters of each filtered supernatant were combined with 20 µL of the 5X methyl red solution in a 96-well plate. The color absorbance of methyl red was measured at 529 nm and 435 nm with pathlength correction (Tecan Infinite Mplex). The 529/435 ratio of the pathlength-corrected absorbance was calculated, and the pH was derived from a standard curve previously generated with SLM pH-adjusted to values between 3.75 and 7.25. For measurement of extracellular pH in SLM at pH 7.0, a phenol red stock (1.2 mg/mL in 100% EtOH) was diluted 1:100 (vol/vol) in Ultrapure water to generate a 5X solution. Eighty microliters of each filtered supernatant were combined with 20 µL of the 5X phenol red solution. The color absorbance was measured at 415 nm and 560 nm with pathlength correction, the 415/560 ratio of the pathlength-corrected absorbance was calculated, and the pH was derived from a standard curve previously generated with SLM pH-adjusted to values between 3.0 and 10.5.

### Extracellular ammonium assays

A NH_4_Cl standard curve (0–32 mM) was generated in SLM for this assay. Fifty microliters of standards and samples were added to a 96-well plate, followed by 100 µL of phenol nitroprusside (Millipore Sigma P6994). The reaction was started with 100 µL of alkaline hypochlorite solution (Millipore Sigma A1727), and the reaction was developed in a fume hood shaking at 300 RPM for one hour. Fifty microliters of the reaction were then diluted into 150 µL of Ultrapure water in a 96-well plate, and the absorbance was measured at 570 nm. The ammonium concentration for each sample was derived from the generated ammonium standard curve.

### Urease assay

A continuous, coupled enzyme assay to assess urease activity from cell lysates was adapted from previously published work (52). To prepare cell lysates, 450 µL of cultures were centrifuged at 21,000 *× g* for five minutes. Supernatant was removed, and cell pellets were stored at −80°C until the day of the assay. Upon thawing, cell pellets were resuspended in 500 µL lysis buffer (10 mM Tris•HCl pH 8.0, 1 mM PMSF, 100 µg/mL lysostaphin, 10 µg/mL DNase) and lysed at 37°C for 30 minutes. Lysed cells were cooled on ice for five minutes and centrifuged at 21,000 *× g* for 10 minutes (Fisher Scientific AccuSpin Micro 17). Three hundred microliters of supernatant were transferred into a mini dialysis device and placed in a tube floater (Slide-A-Lyzer MINI Dialysis 3,500 MWCO, ThermoFisher Scientific Cat. No. 69552). The samples were dialyzed in 1 L dialysis buffer (10 mM Tris•HCl pH 8.0) at room temperature for one hour, repeated three times. The protein concentration of the cell lysates was quantified using the Pierce BCA Protein Assay (Thermo Scientific Cat. No. 23225) according to the manufacturer’s protocol, and cell lysates were diluted to 40 µg/mL in dialysis buffer. The enzyme reaction was mixed in a 900 µL volume, with final concentrations of the following calculated for a final volume of 1 mL: 10 mM Tris•HCl pH 8.0, 2 mM α-ketoglutarate, 0.5 mM NADH, 10 U/mL glutamate dehydrogenase, and 10 µg/mL cell lysate. A negative control lacking cell lysate and a positive control containing 0.1 U/mL of jack bean urease in lieu of cell lysate were also generated. Each of these reaction mixtures was aliquoted 180 µL into each of three wells of a 96-well plate. Reactions were started with 20 µL of 50 mM urea solution, and depletion of NADH was monitored at 340 nm using a 20–30-second kinetic interval over one to two hours (Tecan Mplex). The linear enzymatic rate was used to calculate urease activity. To convert the absorbance rate to moles of urea consumed/min/mg protein, the NADH extinction coefficient of 6,220 M^−1^ cm^−1^ was used to generate moles NADH/min, and this value was divided by two to convert to moles of urea consumed and normalized to total protein content. Technical replicate values were averaged together, and biological replicate variance was used to calculate error.

### RNA purification

LAC WT, LAC ∆*ure*, and LAC ∆*ure* φ11::URE2 strains were grown in four biological replicates in 5 mL of TSB and 18 × 150 mm glass tubes, shaking at 250 RPM and 37°C for 16 hours. The overnight cultures were then subcultured at a 1% dilution into 25 mL of SLM in 125 mL flasks. These cultures were shaking at 250 RPM and 32°C. When cultures reached a 600 nm absorbance of 0.2–0.3 in a cuvette (1 cm pathlength), 15 mL of the cultures were collected in a Falcon tube and centrifuged at 3,200 *× g* at 25°C for 10 minutes in an Eppendorf 5810R centrifuge equipped with a S-4-104 rotor. After decanting the supernatant, cell pellets were quickly resuspended in ice-cold 1X PBS and centrifuged at 21,000 *× g* for one minute. The 1X PBS was removed, and cell pellets were flash frozen on dry ice and stored at −80°C for up to a week. For RNA purification, the RNeasy Mini Kit (Qiagen Cat. No. 74104) was used with the following instructions. Cell pellets were thawed and resuspended in 100 µL 1X Tris-EDTA buffer (diluted from 20X TE Buffer, RNase-free, T11493) then transferred to an autoclaved bead-beating tube (Fisher Scientific Cat. No. 02-681-375) containing approximately 100 µL of 0.1 mm diameter zirconia/silica beads (BioSpec Cat. No. 11079101z). Samples were bead-beaten for 60 seconds (BioSpec Mini-Beadbeater 16), followed by addition of 650 µL of RLT buffer + 1% β-mercaptoethanol and another 60-second period of bead beating. Tubes were centrifuged at 21,000 *× g* for 30 seconds, and 600 µL of supernatant was transferred to a microfuge tube containing 900 µL 100% ethanol, immediately mixed, and 600 µL was loaded onto a RNeasy column. Columns were centrifuged at 14,000 *× g* for 30 seconds, and flow-through was discarded. The loading of the RNeasy column with the supernatant-ethanol mixture was repeated until all the sample had been transferred to the column. Then, 700 µL of RW1 buffer was added to the column, centrifuged, and flow-through discarded. The column was then washed twice with 500 µL RPE buffer, centrifuged, and flow-through discarded. Columns were centrifuged at 14,000 *× g* for two minutes to dry the membrane, and RNA was eluted into a clean microfuge tube with 53 µL DEPC-treated water and centrifuged at 14,000 *× g* for 30 seconds.

### DNase treatment and cDNA synthesis

To 53 µL of purified RNA, 6 µL of 10X DNase buffer and 1 µL of DNase (Turbo DNA-free kit, Invitrogen AM1907) were added and incubated at 37°C for 60 minutes. Then, 7 µL of DNase deactivation buffer was added, mixed, and centrifuged at 10,000 *× g* for 2.5 minutes. Approximately, 55 µL of the DNase-treated RNA was moved to a fresh tube, and the RNA concentration was quantified using the Qubit RNA BR Assay and Qubit 3.0 according to the manufacturer’s instructions. Conversion of 1 µg of RNA to cDNA was performed with the SuperScript IV VILO Master Mix (Invitrogen Cat. No. 11756050) per manufacturer’s directions. Reactions omitting the reverse transcriptase (RT-) were also performed for each sample.

### Reverse transcriptase, quantitative PCR (RT-qPCR)

qRT-PCR reactions were set up using purified RNA as described above, and the qPCR primers were shown in Table S2. PCR reactions were set up according the to the manufacturer’s instructions (Applied Biosystems, PowerTrack SYBR Green Master Mix, Cat. No. A646110). Primers were validated with a minimum confirmed primer efficiency of 95%. Purified genomic DNA was used as a positive control, and RT samples were used as negative controls. The qRT-PCR reactions were run on a BioRad C1000 Touch Thermal Cycler equipped with a CFX96 Real-Time System reader. *C*_*t*_ values were obtained using the BioRad CFX Manager, and fold changes were calculated using the 2^-ΔΔCt^ method, using *gyrB* as the reference gene.

### Intracellular pH assay

The protocol for intracellular pH assessment was adapted from previously published work (31). Standard curve buffers were prepared by mixing different proportions of a 0.1 M citric acid stock and a 0.2 M dibasic sodium phosphate stock as described (dx.doi.org/10.17504/protocols.io.bfydjps6). For buffers at pH 8 and pH 8.5, 0 mL of 0.1 M citric acid stock and 50 mL of 0.2 M dibasic sodium phosphate stock were used. Once mixed, these standard curve buffers were calibrated to a final pH range of pH 5.0–8.5 in 0.5 increments using either hydrochloric acid or sodium hydroxide before adjusting the total volume to 100 mL with water. Cultures were grown overnight in 5 mL of TSB in 18 × 150 mm glass tubes for 16 hours, shaking at 250 RPM and 37°C, in biological triplicate. These cultures were centrifuged at 3,200 *× g* at 25°C for 10 minutes in an Eppendorf 5810R centrifuge equipped with a S-4-104 rotor, and the supernatant was decanted. Cell pellets were then washed in 1 mL of water and centrifuged in 1.8 mL microfuge tubes at 8,000 *× g* for two minutes, and this was completed twice before a final resuspension in 1 mL of water. Cultures were then diluted to an OD = 1.00 (600 nm) in either the sample buffer (20 mM MES pH 4.5, 100 mM NaCl, 10 mM KCl, 5 mM MgSO_4_, 1 mM CaCl_2_) or in the standard curve buffers. Two hundred microliters of samples were added to a 96-well plate, and a kinetic assay monitoring OD (600 nm) and fluorescence (excitation 410 and 470 nm, emission 510 nm, gain 90) every minute was initiated with a Tecan plate reader. After 10 minutes of reads for the samples, 10 µL of 200 mM urea stock in water, or 10 µL of a water control, were added to the samples. After 60 minutes of total assay time, samples were centrifuged, and the pH of the supernatant was quantified as described in the extracellular pH assays section of the methods. After collection of sample data, 200 µL of the standards were added to the plate, monitored for 10 minutes using the above assay parameters, and then 5 µL of a stock solution containing 80 µM nigericin (diluted from a 2 mM stock in 100% ethanol) and 80 µM valinomycin (diluted from a 2 mM stock in 100% dimethyl sulfoxide) was added. The culture density and fluorescence were monitored for 20 minutes post-antibiotic addition. For each well and time point, the 410/470 fluorescence ratio was calculated. The 410/470 nm fluorescence ratio and corresponding buffer pH data were fit to a sigmoidal curve, and this standard curve was used to calculate the intracellular pH of the samples.

## SUPPLEMENTAL MATERIAL

Supplemental figures and table jb.00208-25-s0001.docx Fig. S1 to S8 and Table S1.

ASM does not own the copyrights to Supplemental Material that may be linked to, or accessed through, an article. The authors have granted ASM a non-exclusive, world-wide license to publish the Supplemental Material files. Please contact the corresponding author directly for reuse.
