# Supplementary material for: Urease promotes pH homeostasis and growth of Staphylococcus aureus in skin-like conditions
Source: J Bacteriol. 2025 Sep 8;207(10):e00208-25. doi: 10.1128/jb.00208-25 (PMC12548400; doi:10.1128/jb.00208-25)
Supplement: Supplemental figures and table — Fig. S1 to S8 and Table S1. [file jb.00208-25-s0001.docx]

**Supplemental Information**

**Urease promotes pH homeostasis and growth of *Staphylococcus aureus* in skin-like conditions.**

Flavia G. Costa^1^, Alexander R. Horswill^1,2*^

^1^Department of Immunology and Microbiology, University of Colorado Anschutz Medical Campus, Aurora, Colorado, United States of America.

^2^Department of Veterans Affairs, Eastern Colorado Healthcare System, Aurora, Colorado, United States of America.

*Corresponding author

Email: alexander.horswill@cuanschutz.edu

**Table S1. Details on plasmid construction.** A description of the cloning approach and list of primers used to generate each plasmid used in this work are listed below.

| Plasmid Name | Genotype | Description | Primers |
| --- | --- | --- | --- |
| pURE1 | pIMAY::  ∆*ureABCEFGD* | Fragments upstream and downstream of the urease operon were amplified from AH1263 and ligated into the pIMAY vector using the SacI and SalI restriction sites, and a KpnI site was used to join the two fragments together. | Upstream fragment:  For:  CCCgagctcCGAAATATAAGGGTTAATCGCTG  Rev: CCCggtaccACCCCCAATTTCATATTAGATAC  Downstream fragment:  For: CCCggtaccgACTAGTGTACCTTGTTTCAAGC  Rev:  CCCgtcgacATTGGTAATGTCCCAATGCATG |
| pURE2 | pLL29erm::  P*_ure_*_*ureABCDEFGD* | The urease operon and its native promoter were amplified from AH1263 and ligated into the pLL29erm vector using the EcoRI and SacI restriction sites. | Fragment:  For: NNNNgaattcATCTATTATCTTCAAATCTTTCACCTT  Rev: NNNNgagctcTTAATATTTTCTTAGAAAATCAAGTTTACGA |
| pURE3 | pIMAY::∆ureT | Fragments upstream and downstream of the putative urea transporter (SAUSA300_2237) were amplified from AH1263 and ligated into the pIMAY vector using the KpnI and SacI restriction sites, and an EcoRI site was used to join the two fragments together. | Upstream fragment:  For:  GTAGggtaccGCTGGAATATCTAAATGTTT  Rev:  GTCAgaattcAAGCACATCTATTATCTTCA  Downstream fragment:  For:  GTAGgaattcGATGCTTGATAGTTAAATCA  Rev:  GTAGgagctcGCTTTACTTTTCTCTGTTT |
| pURE4 | pLL29erm::  P*_ureT__ureT* | The putative urea transporter and its native promoter were amplified from AH1263 and ligated into the pLL29erm vector using the BamHI and SalI restriction sites. | Fragment:  For:  CAATggatccCTCGTTGTGTAAAATGCAAA  Rev:  GCATgtcgacGGTGATATTCAAACAATATTAGG |
| pURE13 | pCM28::  P*_cons__ureD* | The constitutive promoter was amplified from the pHC125 vector, and *ureD* was amplified from AH1263. The fragments were ligated into pCM28 linearized with NheI and EcoRI using Gibson assembly. | Promoter fragment:  For:  acagctatgacatgattacgATCTCGAGATCTGCAAGATC  Rev:  agtgtcgcgtAGGTATTTGTGTACCTGG  *ureD* fragment:  For:  acaaatacctACGCGACACTTTACTCAAAG  Rev:  ggtcgactctagaggatccgAGTCTTTTTATTTTAATATTTTCTTAGAAAATCAAG |

**Table S2. qPCR primers.** A list of qPCR primers used in Fig. S1.

| **Primer Name** | **Sequence** |
| --- | --- |
| gyrB_F | AATTATTCTCAGAGCACTTTGAAC |
| gyrB_R | TACATCTAACGCTGATTTACGA |
| SAUSA300_2237_F | ATCAGGGATTATTCGGTTATAACT |
| SAUSA300_2237_R | TCAAGCAATGTTGTTGTACCTA |
| ureA_F | ACGAGAGCAAGACAAATTAATG |
| ureA_R | ACCTTCTAATAATTCATCGCTGA |
| ureD_F | AGCCTTCAAGTATCAATATATGCA |
| ureD_R | AGAGCCATAATGAGAGTAATGTT |
| sarR_F | GCTCAGAGTTCAAACCTTACT |
| sarR_R | CAATAACTGTTCTTTCGTCTTGT |

**
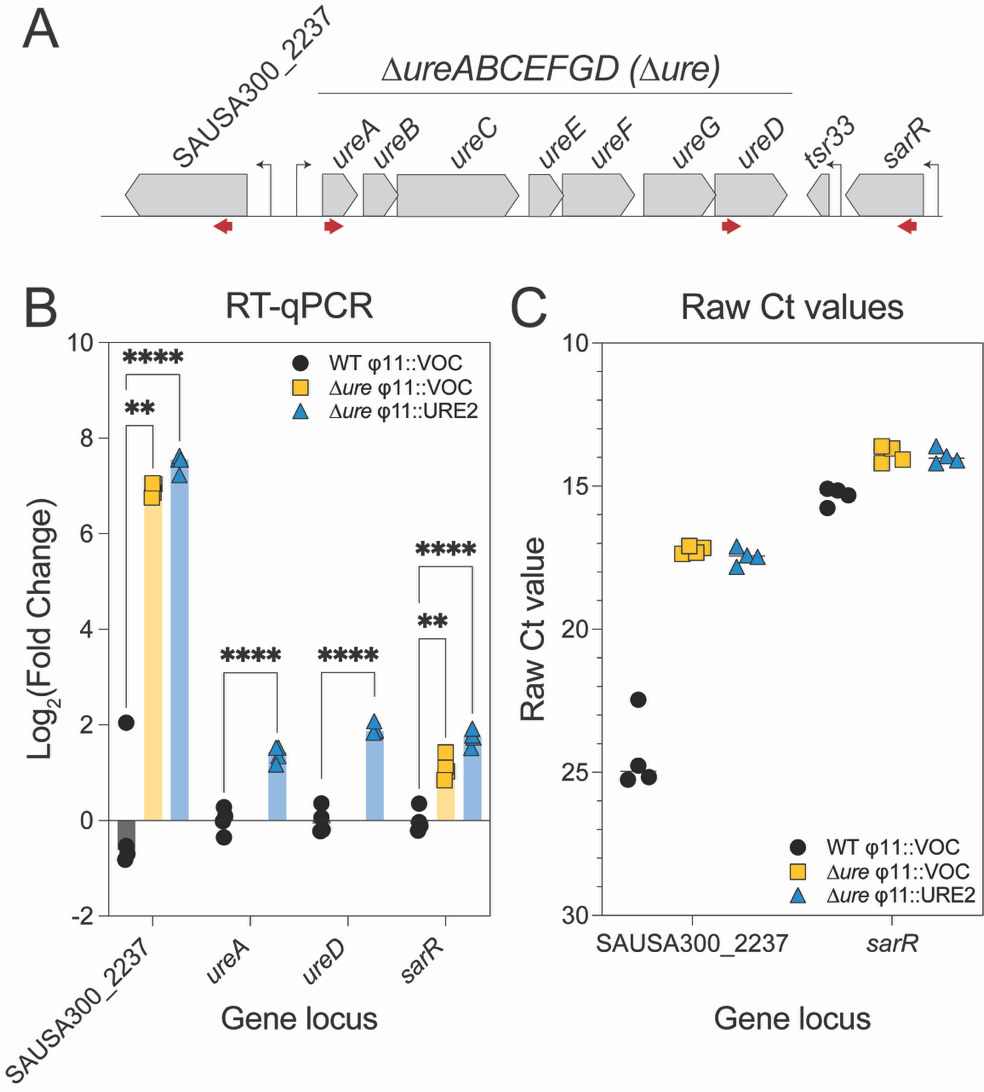
**

**Figure S1. Clean deletion of the urease operon results in artificial upregulation of the putative urea transporter.** (A) The genetic locus of the urease operon and surrounding genes are depicted. The red bars indicate the regions amplified by primers used in RT-qPCR experiments. (B) The log_2_-fold change of the putative urea transporter (SAUSA300_2237), *ureA*, *ureD*, and *sarR* in the WT, ∆*ure*, and ∆*ure* ϕ11::*ure* strains, as compared to transcription of gyrB and the WT strain using the 2^∆∆Ct^ method. (C) The C_t_ values from the RT-qPCR experiment shown in (B), demonstrating comparable C_t_ values for SAUSA300_2237 and the *sarR* transcripts. Statistics were completed with one-way ANOVA. ** = p < 0.01, **** = p < 0.0001.

**Figure S2. Input CFU/mL for data in Figure 3.** CFU/mL calculated from dilution plating of input cultures. Statistics were calculated using an unpaired t-test with a p-value of 0.05 cutoff, ns = not significant.


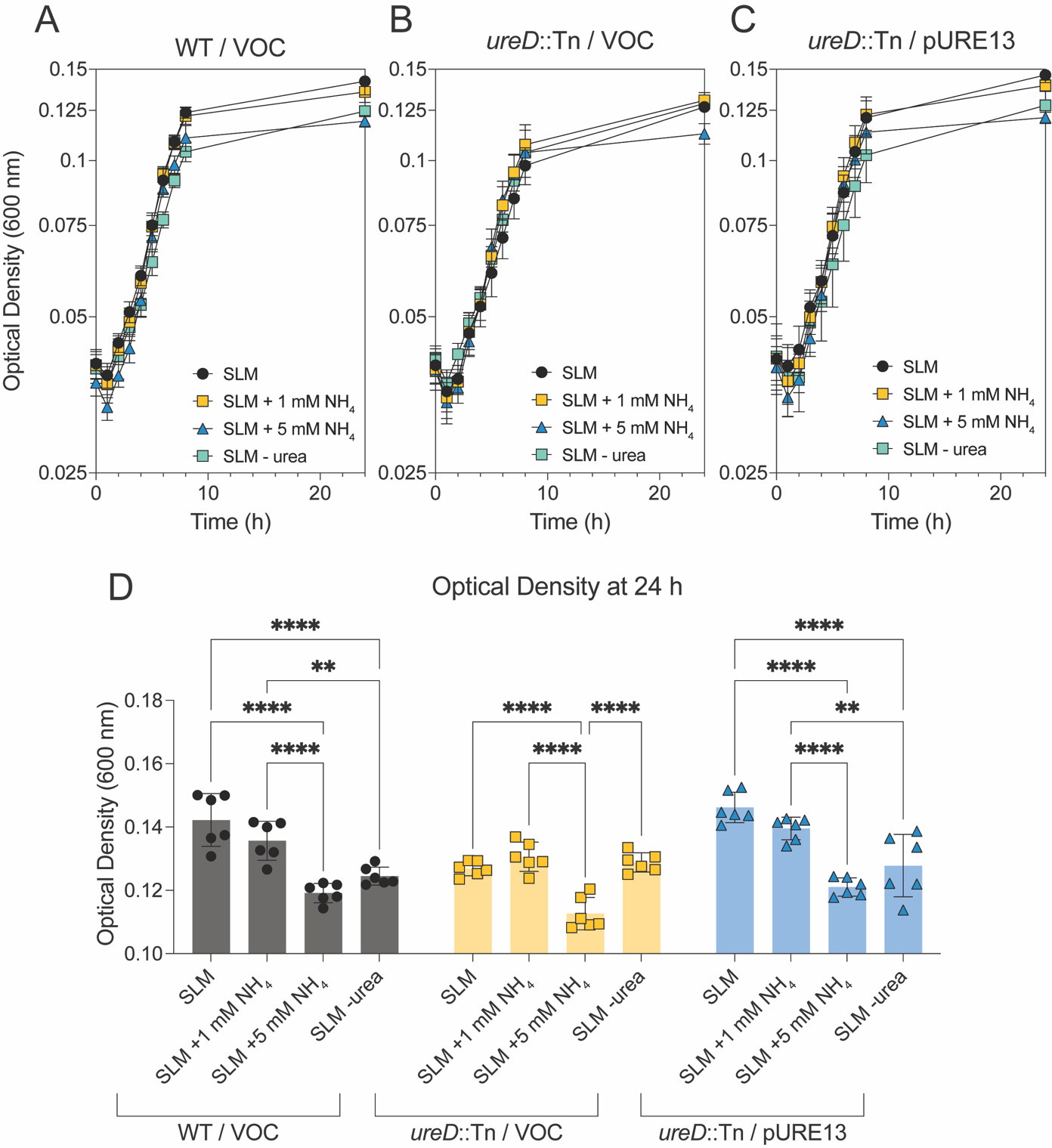


**Figure S3. Comparison of data from Figure 4 by strain.** Strains WT / VOC (A), *ureD*::Tn / VOC (B), *ureD*::Tn / pURE13 (C) grown in SLM (black circles), SLM + 1 mM NH_4_ (yellow squares), SLM + 5 mM NH_4_ (blue triangles), and SLM – urea (turquoise squares). (D) Comparison of OD after 24 hours of growth in SLM with each strain, across conditions. Addition of 5 mM NH_4_ to SLM significantly reduced optical density of all three strains after 24 hours. Statistics were calculated using two-way ANOVA with a post-hoc Tukey test comparing differences within each strain, across conditions. ** = *p* < 0.01, **** = *p* < 0.0001.


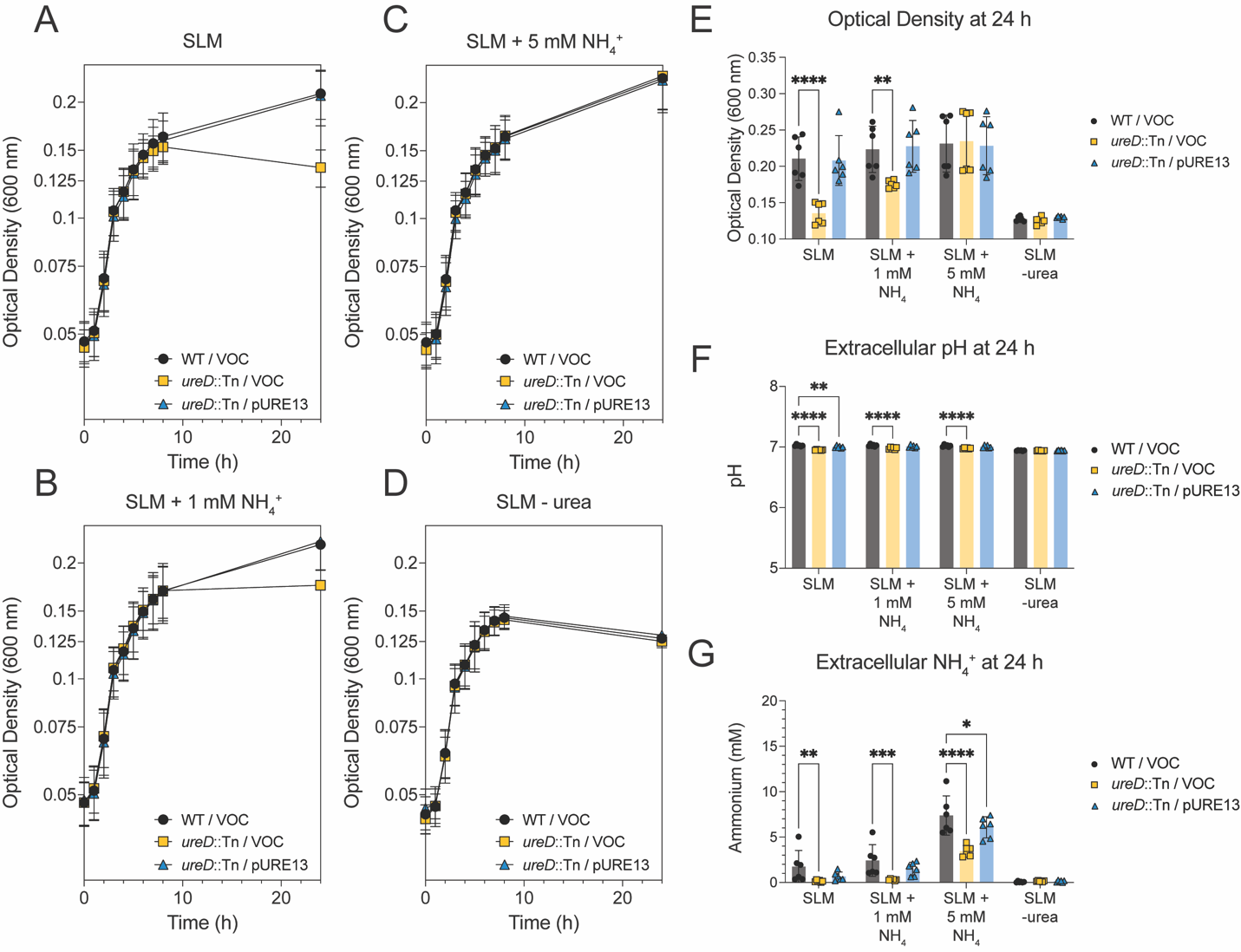


**Figure S4. The *ureD*::Tn growth defect is complemented by the addition of ammonium at neutral pH.** Growth of WT / VOC (black circles), *ureD*::Tn / VOC (yellow squares), and *ureD*::Tn / pURE13 (blue triangles) in (A) SLM pH 7.0, (B) SLM pH 7.0 + 1 mM NH_4_, (C) SLM pH 7.0 + 5 mM NH_4_, and (D) SLM pH 7.0 -urea. (E) A bar graph of culture absorbance at 24 hours from growth curves in (A-D). (F) A bar graph of culture supernatant pH at 24 hours from growth curves in (A-D). (G) A bar graph of ammonium concentration in culture supernatant at 24 hours from growth curves in (A-D). Data shown are from two experiments, each experiment in biological triplicate. Statistics were calculated using two-way ANOVA, with a post-hoc Dunnet’s test. * = *p*<0.05, ** = *p*<0.01, *** = *p*<0.001, **** = *p*<0.0001.


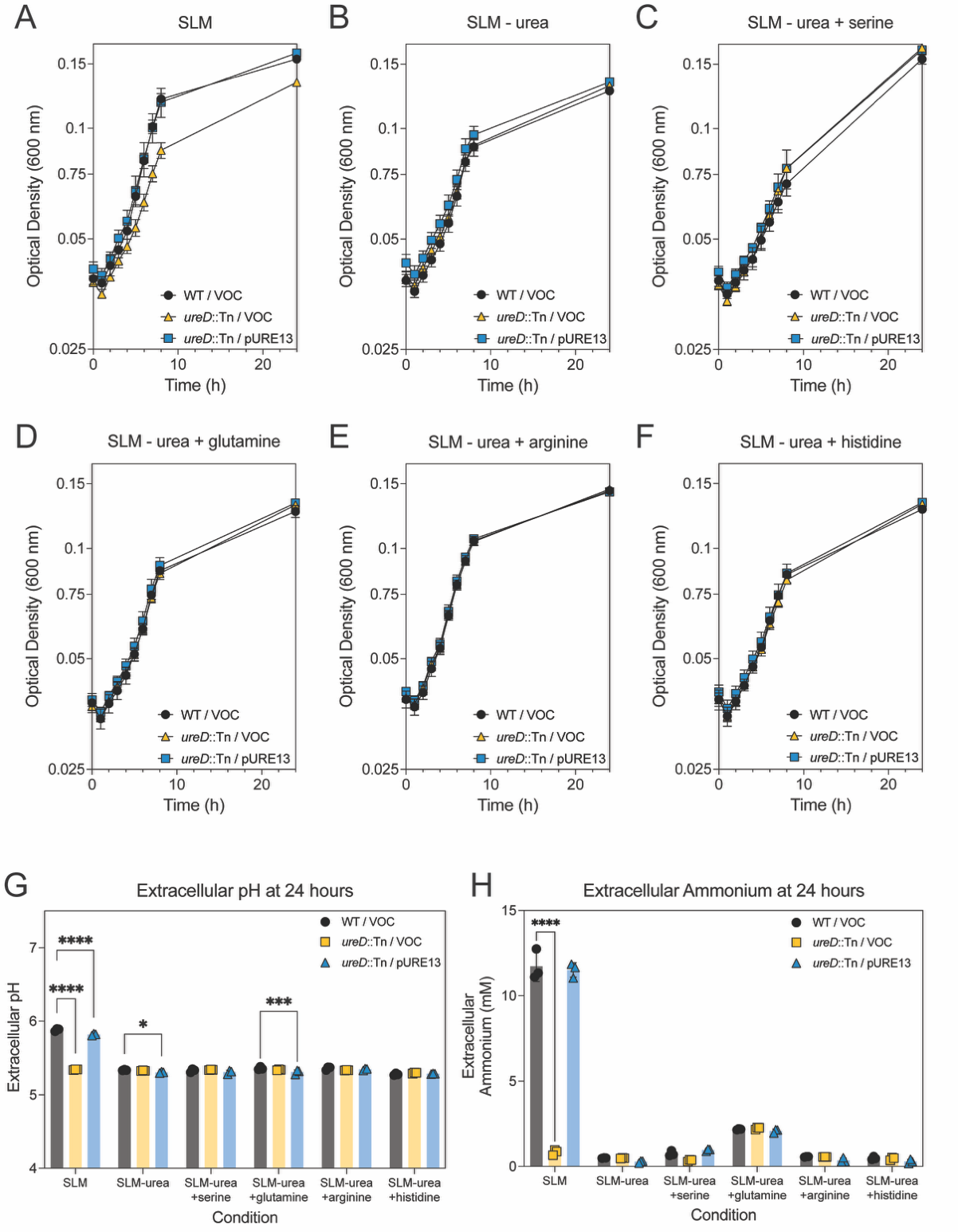


**Figure S5. Data including *ureD*::Tn mutant and complement strain for experiments in Figure 5.** Growth of WT / VOC (black circles), *ureD*::Tn / VOC (yellow squares), and *ureD*::Tn / pURE13 (blue triangles) in (A) SLM, (B) SLM no urea, (C) SLM no urea and 10 mM serine, (D) SLM no urea and 10 mM glutamine, (E) SLM no urea and 10 mM arginine, (F) SLM no urea and 10 mM histidine. At 24 hours, (G) The pH of the culture media was assessed using the methyl red assay, and (H) the ammonium concentration in the culture media was assessed using the phenol hypochlorite assay. Statistics in (G-H) were calculated with a two-way ANOVA and a post-hoc Dunnet’s test comparing to WT / VOC. * = *p* < 0.05, *** = *p* < 0.001, **** = *p* <0.0001.


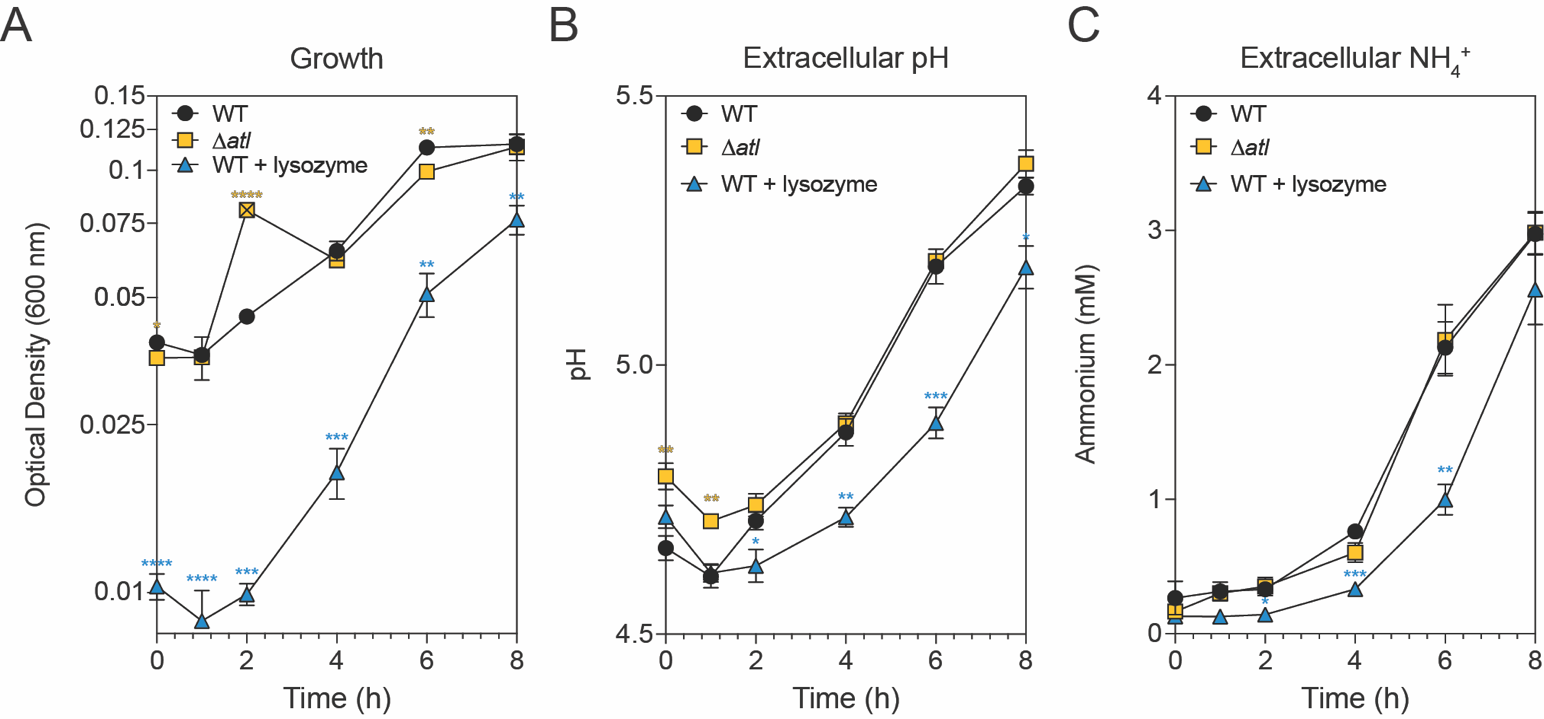


**Figure S6. Autolysis does not contribute to extracellular ammonium generation.** (A) Growth of WT, ∆*atl,* and WT pretreated with lysostaphin grown in SLM. The datapoint marked with an X denotes cell aggregation observed in the ∆*atl* strain that is not observed at other timepoints or with other strains. (B) Extracellular pH and (C) Extracellular ammonium measured from the culture supernatant. Cultures were grown in biological triplicate. Statistics were calculated by two-way ANOVA with the Geisser-Greenhouse correction, with matched values stacked into a sub column. Post-hoc Dunnet’s test was performed with individual variances computed for each sub column. * = *p* < 0.05, ** = *p* < 0.01, *** = *p* < 0.001, **** = *p* < 0.0001.


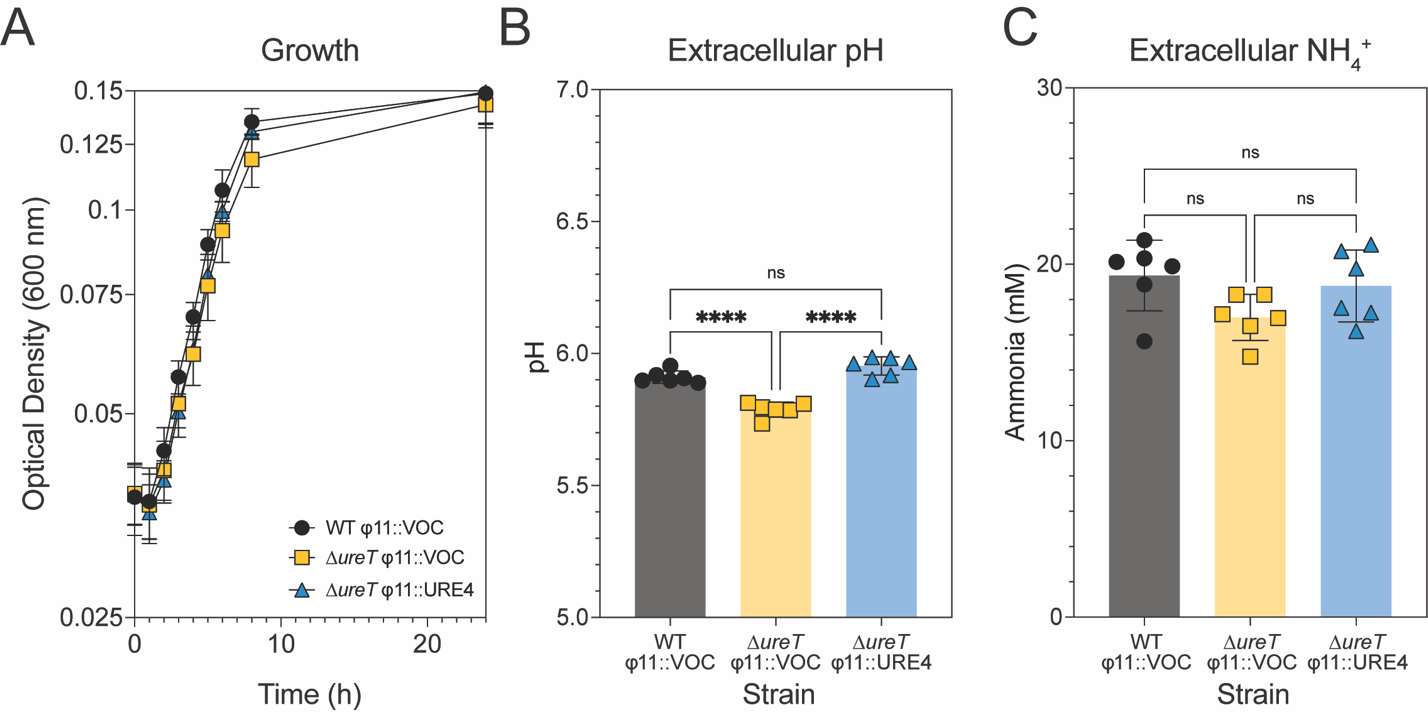


**Figure S7. UreT phenotype at 24 hours.** (A) WT, *∆ureT* mutant, and complementation strains were growth in SLM for 24 hours. (B) Extracellular pH was measured at 24 hours using the methyl red assay. (C) Extracellular ammonium was measured at 24 hours using the phenol hypochlorite assay. Statistics for (B-C) were calculated using a one-way ANOVA and Tukey’s multiple comparisons test. **** = *p* < 0.0001.


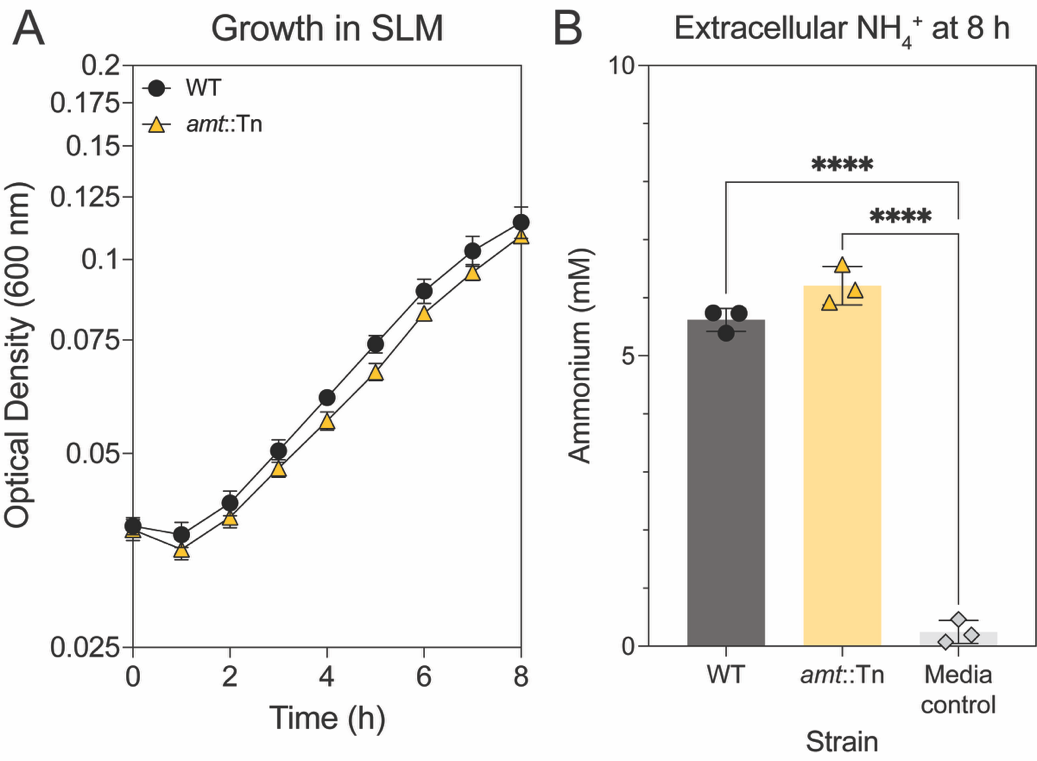


**Figure S8. Putative ammonium transporter Amt does not contribute to extracellular ammonium generation.** Transposon mutant *amt::*Tn was obtained from the Nebraska Transposon Mutant Library and backcrossed into strain LAC using phage transduction. (A) WT and *amt*::Tn cultures were grown in SLM for 8 hours. (B) At 8 hours, culture supernatants were harvested to measure extracellular ammonium generation. No significant difference was found between WT and *amt*::Tn in extracellular ammonium generation. Statistics for (B) were calculated using one-way ANOVA, followed by a post-hoc Tukey’s test with a single pooled variance. **** = *p* < 0.0001.
